# Pharmacokinetic study of Tangwang Mingmu granule for the management of diabetic retinopathy based on network pharmacology

**DOI:** 10.1080/13880209.2021.1979051

**Published:** 2021-09-30

**Authors:** Yucheng Wang, Beibei Xue, Xiaoli Wang, Qilong Wang, Erwei Liu, Xiaopeng Chen

**Affiliations:** State Key Laboratory of Component-based Chinese Medicine, Tianjin University of Traditional Chinese Medicine, Tianjin, China

**Keywords:** Chemical profiling, marker, herb medicine, luteolin, formononetin

## Abstract

**Context:**

Tangwang Mingmu granule (TWMM), a traditional Chinese medicine, has been widely used in the treatment of diabetic retinopathy (DR), the most common microvascular complication in diabetes mellitus.

**Objective:**

To establish a method to select target compounds from herbs for a pharmacokinetic study using network pharmacology, which could be applied in clinical settings.

**Materials and methods:**

First, UPLC/Q Exactive Q-Orbitrap and GCMS 2010 were used to determine the non-volatile and volatile ingredients of TWMM. Based on the identified compounds, network pharmacology was used to screen the key compounds and targets of TWMM in the treatment of DR. Based on the compound-target-pathway network and identification of components emigrant into blood, the potential compound markers *in vivo* were chosen. Then, Sprague-Dawley (SD) rats were administrated of TWMM at a 9.6 g/kg dose to investigating pharmacokinetic parameters using the UPLC-QQQ-MS.

**Results:**

Ninety and forty-five compounds were identified by UPLC-MS and GC-MS, respectively. Based on the network pharmacology, nine compounds with a degree value above 15 were screened and implied that these compounds are the most active in DR treatment. Moreover, criteria of degree value greater than 7 were applied, and PTGS2, NOS2, AKT1, ESR1, TNF, and MAPK14 were inferred as the core targets in treating DR. After identification of components absorbed into blood, luteolin and formononetin were selected and used to investigate the pharmacokinetic parameters of TWMM after its oral administration.

**Conclusions:**

The reported strategy provides a method that combines ingredient profiling, network pharmacology, and pharmacokinetics to determine luteolin and formononetin as the pharmacokinetic markers of TWMM. This strategy provides a clinically relevant methodology that allows for the screening of pharmacokinetic markers in Chinese medicines.

## Introduction

Pharmacokinetic research plays a critical role in assessing drug clinical dosing parameters, side effects, and treatment mechanisms *in vivo* (Ding et al. 2019; Liu et al. 2020). To understand the pharmacokinetic parameters of absorption, distribution, metabolism, and excretion (ADME) of a drug product, the high-content-ingredients that emigrate into the circulatory system are generally selected as target compounds in a pharmacokinetic study (Jiang et al. 2020; Liu et al. 2020). In certain instances, the activity of the high content ingredients does not match the clinical application of the herbal medicine, resulting in difficulties in obtaining pharmacokinetic indicators to guide clinical use. Occasionally, broad action(s) of detected compounds, such as immune-stimulatory and antioxidative activities are reported without provision of any proof to support their specific clinical contribution in the herb or prescription medicine for the management of diseases such as DR (Yuan et al. 2015; Liu et al. 2019). For example, the clinical symptoms of chronic nephritis have been reported to be alleviated by shenyanyihao oral solution. Ten high-content compounds including stachyBine, danshensu, chlorogenic acid, protocatechuic acid, plantamajoside, aesculetin, isoquercitrin, ferulic acid, baicalin, and baicalein were selected for a pharmacokinetic study which resulted in little clinical relevance (Jiang et al. 2020). Similarly, Liu et al. (2020) reported that Chaihu-Shugan-San could be used to treat depression. In this study, nine high content ingredients were selected as markers and determined in plasma samples. However, most of these compounds were not interrelated to the treatment of depression. Thus, establishing a method that can determine clinically relevant compounds in pharmacokinetic studies of complex drugs is warranted.

Network pharmacology analyzes the network of the biological system and selects specific signal nodes to design multi-target drug molecules based on the theory of systems biology. It can demonstrate compound-target-pathway networks, which can be used to explore the mechanism of various compounds in treating multi-target diseases from a perspective of systems biology and biological network balance (Yang et al. 2019). In addition, this comprehensive analysis can be applied in the screening of active ingredients and therapeutic targets as well as in determining the compound mechanism of action (Pan et al. 2020; Zhang et al. 2020). Zhang et al. (2021) reported that Shuang-Huang-Lian water extract (SHL) considerably improves the symptoms of upper respiratory tract infection. In their study, baicalin, sweroside, chlorogenic acid, forsythoside A, and phillyrin were selected as the potential active compounds through network pharmacology. Consequently, H1N1-infected mice were administered these five compounds to verify their therapeutic effects. They found that these five compounds had the same therapeutical effects as SHL, causing the release of cytokines such as TNF-α, IL-1β, and IL-6, and ultimately contributing to an increased survival rate. Therefore, network pharmacology is capable of identifying active ingredients responsible for pharmacological activity in mixed medicines.

DR is one of the most serious complications of diabetes mellitus, eventually leading to vision loss and even blindness if not managed (Sabanayagam et al. 2019). Since visual sense involves all aspects of our daily lives, the deterioration of vision is likely to seriously affect the quality of life in these patients. However, the pathogenesis of DR is not clear, and current symptomatic therapy of the disease is ineffective (Whitehead et al. 2018; Nawaz et al. 2019). Furthermore, the available drugs for the treatment of DR are deficient and unilateral (Stitt et al. 2016). The commonly used treatment for DR, an intraocular injection containing anti-vascular endothelial growth factor (VEGF), neither works in early DR nor restrains the development of DR (Dulull et al. 2019). In addition, multiple intraocular injections raise the risk of serious intraocular infection. Other treatments such as fundus laser photocoagulation involve the risk of destroying the normal function of the retina (Shalchi et al. 2020; Mones et al. 2021). Currently, all the available treatment options for DR may result in poor treatment compliance and disease prognosis (Fajnkuchen et al. 2020). Therefore, novel orally administered therapy is an urgent need in attempting to manage DR.

Herbal medicine has certain advantages in the treatment of chronic diseases with complex mechanisms (Li and Weng 2017). TWMM, an herbal product derived from Mimenghuafang, has been applied clinically for 20 years as a decoction (Song et al. 2015). It contains seven herbs, i.e., *Astragalus propinquus* Schischkin (Leguminosae), *Coptis chinensis* Franch. (Ranunculaceae), *Buddleja officinalis* Maxim. (Scrophulariaceae), *Cinnamomum cassia* (L.) J.Presl (Lauraceae), *Prunus mume* (Siebold) Siebold & Zucc. (Rosaceae), *Ligustrum lucidum* W.T.Aiton (Oleaceae) and *Leonurus japonicus* Houtt (Lamiaceae). Clinical applications show that TWMM can significantly improve the vision of patients with DR, alleviate clinical symptoms such as asthenopia, and improve the state of blood vessels in the fundus (Chen et al. 2017). TWMM has been shown to possess a curative effect in the management of DR. The retinal protective effects of TWMM in diabetic rats was postulated to result from the upregulation of SOCS3 expression, inhibition of the JAK/STAT signalling pathway, and further inhibition of VEGF expression (Chen et al. 2017). Furthermore, TWMM was shown to restore the ratio of VEGFR-1/VEGFR-2, thus maintaining the normal activities of endothelial cells and inhibiting the abnormal proliferation of capillaries in the retina (Song et al. 2015).

In this study, identification of active compounds that are responsible for pharmacological activity in medicinal mixtures and network pharmacology was applied before embarking on a pharmacokinetic study. TWMM was studied as a model and the observed results provide a theoretical foundation for the clinical application of TWMM. Furthermore, markers that were screened by the established method were successfully applied in the pharmacokinetic study and possessed similar clinical indications of the herbal mixture. Overall, this study revealed the pharmacological properties of drugs in a clinically relevant manner and provided a reference for the pharmacokinetic study of multi-component drug mixtures.

## Materials and methods

### Materials and reagents

The TWMM was provided by Beijing HanDian Pharmaceutical Co., Ltd. (Beijing, China). Chlorogenic acid, linarin, luteolin, jaspolyside, tolbutamide, stachydrine, magnoflorine, calycosin-7-*O*-β-d-glucoside, stepharanine, jatrorrhizine, epiberberine, columbamine, ononin, berberine, palmatine, berberrubine, calycosin, formononetin, 2α-hydroxyoleanic acid, hydroxytyrosol, salidroside, luteolin-7-*O*-glucoside, verbascoside, specnuezhenide, oleuropein, physcion, oleonuezhenide, astragaloside IV, astragaloside II, isoastragaloside II, astragaloside I, luteolin, formononetin, and tolbutamide (purity ≥ 98%) were purchased from Shanghai Yuanye Pharmaceutical Technology Co., Ltd and Sichuan Weikeqi Pharmaceutical Technology Co., Ltd. Acetonitrile and formic acid both in HPLC grade were obtained from Fisher Scientific (Fair Lawn, NJ, USA). Ultrapure water was prepared using a Milli-Q water purification system (Millipore, Bredford, MA, USA). Other reagents were all analytical grade.

### Preparation of standard and sample solutions

#### Preparation for qualitative analysis

Ten mg of each standard was dissolved in 10 mL of methanol solution. Next, an equal volume of stock solution was mixed in a 10 mL volumetric flask to acquire a mixed standard solution with a certain concentration. Samples of TWMM (20 mg) were added to 10 mL of 70% methanol for UPLC-MS and n-hexane for GC-MS analyses, respectively. The samples were exposed to ultrasound extraction for 15 min and centrifuged at 15,000 *g* for 10 min at 4 °C, followed by subsequent collection of the supernatants. All the solutions were stored at −20 °C before the experiment.

#### Preparation for pharmacokinetic analysis

Analytical standards (10 mg) of oleuropein, chlorogenic acid, formononetin, verbascoside, linarin, luteolin, jaspolyside, specnuezhenide, and tolbutamide (internal standard, IS) were dissolved in a 10 mL volumetric flask with methanol. An appropriate volume of solution containing oleuropein, chlorogenic acid, formononetin, verbascoside, linarin, luteolin, jaspolyside, and specnuezhenide solution was diluted to obtain a stock solution. Subsequently, the mixture was serially diluted to prepare the reference working solutions and then tolbutamide was added to achieve an IS concentration of 5 μg/mL.

### Analytical conditions

#### UPLC-MS conditions for identification of non-volatile components of TWMM

The identification of non-volatile components was determined using a UPLC/Q Exactive Q-Orbitrap system (Thermo Fisher Scientific, USA). Chromatographic separation was performed on a 1.8 µm HSS T3 analytical column (100 mm × 2.1 i.d.). The mobile phase was composed of 0.1% (*v/v*) acetic acid in water (A) and acetonitrile (B) under the following gradient conditions: 0–30 min, 8–20% B; 30–35 min, 20–25% B; 35–45 min, 25–80% B; 45–45.5 min, 80–8% B; 45.5–49 min, 8–8% B. The flow rate was set at 0.4 mL/min and the temperatures of the autosampler and analytical column were maintained at 35 °C and 4 °C, respectively. In addition, the HESI of the Q-Orbitrap mass spectrometer was set to both negative and positive ionisation modes. The ion source parameters were as follows: spray voltage, +2.9 kV/–2.8 kV; auxiliary gas rate, 10 L/h; sheath gas rate, 35 L/h; auxiliary gas temperature, 400 °C; capillary temperature, 350 °C; normalised collision energy, 10/20/40 V, and mass range, 150–1500 *m/z*.

#### GC-MS conditions for identification of volatile components of TWMM

The profiling of volatile components of TWMM was performed on a Shimadzu GCMS 2010 solution (Shimadzu, Japan). Separation was conducted on a DB-17 column (30 m × 0.25 mm × 0.25 μm). The oven temperature program setting is shown in Table 1. Helium was used as the carrier gas at a flow rate of 1.4 mL/min. The ion source and interface temperatures were set at 230 °C and 250 °C, respectively.

**Table 1.** The oven temperature program of GCMS–QP 2010.

Temperature (°C)	Duration (min)	Flow rate (°C/min)
40–40	3	0
40–106	11	6
106–142.6	12.2	3
142.6–142.6	1	0
142.6–180	6.2	6
180–200	5	4
200–235	17.5	2

#### Network pharmacology

All targets of all compounds constituting TWMM were screened by using the Traditional Chinese Medicine Systems Pharmacology Database (TCMSP, http://lsp.nwu.edu.cn/tcmspsearch.php), which provided the chemical compounds and their related target proteins. Related targets of DR were predicted and screened using the Comparative Toxicogenomics Database (CTD, http://ctdbase.org/), Online Mendelian Inheritance in Man database (OMIM, https://omim.org/), and DrugBank Database (DBD, https://www.drugbank.ca/). The intersection targets were imported into Venny 2.1.0 software (https://bioinfogp.cnb.csic.es/tools/venny/index.html). The protein-protein interaction network (PPI) of intersection targets was created using the STRING Database platform (http://string-db.org/, ver. 11.0) with medium confidence (0.4) to remove the isolated target proteins. Furthermore, the drug-disease crossover genes were annotated and visualised using the Ingenuity Pathway Analysis software (ver. 2019, Redwood City, CA, USA). Lastly, the compound-target-pathway network was established using the Cytoscape interaction network visualisation software (http://cytoscape.org/, ver. 3.5.2).

#### UHPLC-MS/MS conditions for pharmacokinetics

Chromatographic separation was performed using an ACQUITY UPLC H-Class system equipped with a 1.8 μm HSS T3 analytical column (100 mm × 2.1 i.d., Waters Corporation, Milford, USA). The mobile phase consisted of 0.1% (*v/v*) aqueous formic acid (A) and acetonitrile (B) under the following gradient conditions: 0–2 min, 8–13% B; 2–7 min, 13–50% B; 7–9 min, 50–95% B; 9–11 min, 95–95% B; 11–12.5 min, 95–8% B; 12.5–15 min, 8–8% B. The flow rate was set at 0.3 mL/min and the column and autosampler temperatures were maintained at 35 °C and 15 °C, respectively. ESI source was set to negative ionisation mode while the analysis was performed using multiple reaction monitorin. Ion spray voltage (3000 V), capillary temperature (450 °C), and the source parameters of the nine compounds are shown in Table 2.

**Table 2.** Mass spectra properties of 8 compounds and tolbutamide (IS).

Compound	Parention *(m/z)*	Daughterion (*m/z*)	CV (V)	CE (V)
Oleuropein	539.22	138.97	2	28
Chlorogenic acid	353.13	190.99	2	20
Formononetin	267.11	251.97	74	20
Verbascoside	623.24	160.94	2	40
Linarin	591.21	283.03	94	18
Luteolin	285.08	132.95	2	32
Jaspolyside	403.16	223.04	2	12
Specnuezhenide	685.27	523.16	82	20
Tolbutamide	269.14	169.93	74	20

#### Data analysis

The data obtained from the UPLC/Q-Orbitrap MS was imported into Xcalibur 4.0 software for analysis. Furthermore, the constituents acquired using GCMS-QP 2010 were profiled by comparison using the National Institute of Standards and Technology (NIST) database. The pharmacokinetic data were processed through MassLynx 4.1, and DAS 2.0 software was used to calculate the pharmacokinetic parameters according to the compartment model.

### Method validation for pharmacokinetics

Method specificity was assessed by comparing the chromatograms of blank plasma, blank plasma spiked with oleuropein, chlorogenic acid, formononetin, verbascoside, linarin, luteolin, jaspolyside, specnuezhenide, and IS, and plasma after oral administration of TWMM. The calibration curves were assessed at eight concentration levels using the correlation coefficient (*r*). The lower limit of quantification (LLOQ) could fulfil the analytical requirements and achieved reliable accuracy and precision with a signal-to-noise ratio (S/N) ≥ 10. To determine intra- and inter-day precision and accuracy, six replicate quality control (QC) samples at three concentrations were prepared. Inter- and intra-day precision was evaluated by determining relative standard deviation (RSD) values, while accuracy was expressed in terms of the relative error (RE). The extraction recoveries were determined using three concentration levels and calculated by comparing the peak area of extracted samples with post-extracted spiked samples. Subsequently, matrix effects were assessed using the peak area of post-extracted spiked samples contrasted with QC samples at the three concentration levels. The stability test of QC samples at the three concentration levels was as follows: storage at 25 °C for 4 h, storage in an autosampler for 12 h, freeze-thaw cycle for three times, and storage at −80 °C for 7 d.

### Pharmacokinetic study

Six male SD rats (average weight: 220 ± 10 g) were acquired from Beijing Vital-River Laboratory Animal Technology Co., Ltd. All rats were acclimatized to an environmentally controlled laboratory for a week. Following this period, the rats were exposed to fasting conditions for 12 h but were allowed free access to water before the experiment. The rats were orally administered TWMM at a dose of 9.6 g/kg. Blood samples (300 µL) were collected into heparinized centrifuge tubes from the fossa orbitalis at pre-dose and 0.03, 0.08, 0.17, 0.25, 0.5, 1, 2, 4, 8, 12, 24, and 36 h intervals. The samples were immediately centrifuged at 6000 *g* for 10 min at 4 °C and the supernatants were transferred to clean centrifuge tubes.

Each 100 µL plasma sample was mixed with 20 µL of methanol, 20 µL of IS (5 µg/mL), and 600 µL acetonitrile. The mixtures were centrifuged at 15,000 *g* for 10 min at 4 °C after being vortexed for 3 min. The obtained supernatants were transferred to clean 1.5 mL centrifuge tubes, followed by evaporation under a milt nitrogen stream. Next, the obtained residues were individually redissolved in 100 µL of methanol and centrifuged at 15,000 *g* for 10 min at 4 °C after vortexed for 3 min. Lastly, 10 µL of individual supernatant was injected for analysis.

### Molecular docking

The 3D structures of luteolin and formononetin were obtained from the ZINC database (http://zinc.docking.org/). The conformation of the top 5 proteins screened by network pharmacology was collected from the Protein Data Bank (PDB) database: PTGS2 (PDB ID: 5IKR), NOS2 (PDB ID: 4NOS), AKT1 (PDB ID: 3CQW), ESR1 (PDB ID: 1XP9), and TNF (PDB ID: 2AZ5). The CDOCKER program of Discovery Studio 2019 was applied to investigating molecular docking parameters after molecule and protein preparing procedures. Then, PyMol 2.4.0 was used to visualize and verified the result of molecular docking.

### Assay of intracellular reactive oxygen species (ROS)

The generation of ROS was detected using dichloro-dihydro-fluorescein diacetate (DCFH-DA, reactive oxygen species assay kit, Solarbio, Beijing) as per manufacturer instructions. In this study, we seeded HUVEC cells in a 96-well plate at 7 × 10^3^ per well. The cells were treated with luteolin (20, 10, 5 µM) or formononetin (40, 20, 10 µM) for 2 h followed by 30 mM high glucose (HG) for 24 h and incubated for 20 minutes with DCFH-DA (10 mM) at 37 °C. DCF fluorescence was assessed at F488/525 nm by using a bioassay multi-detection fluorescent plate reader (Tecan Spark, Switzerland).

### Luciferase reporter assay

DH5a competent cells (1 × 10^6^) were seeded into 6-well plates. When cell confluence reached about 70%, cells were co-transfected with pGL4.37, pGL4.75 following the manufacturer’s instructions (Lipofectamine RNAiMAX, Invitrogen, USA). Luciferase assays were performed with the dual-luciferase reporter assay system (Promega, Madison, WI, USA) according to the manufacturer’s instructions. Luminescent signals were quantified by a luminometer (Glomax, Promega, Madison, WI, USA), and each value from the firefly luciferase construct was normalized by Renilla luciferase assay.

## Results and discussion

To determine pharmacokinetic markers, we comprehensively performed chemical profiling and then applied network pharmacology to screen key compounds and targets for the treatment of DR using TWMM.

### Qualitative analysis

#### UPLC-Q-Orbitrap-based screening and identification

**Figure 1.**
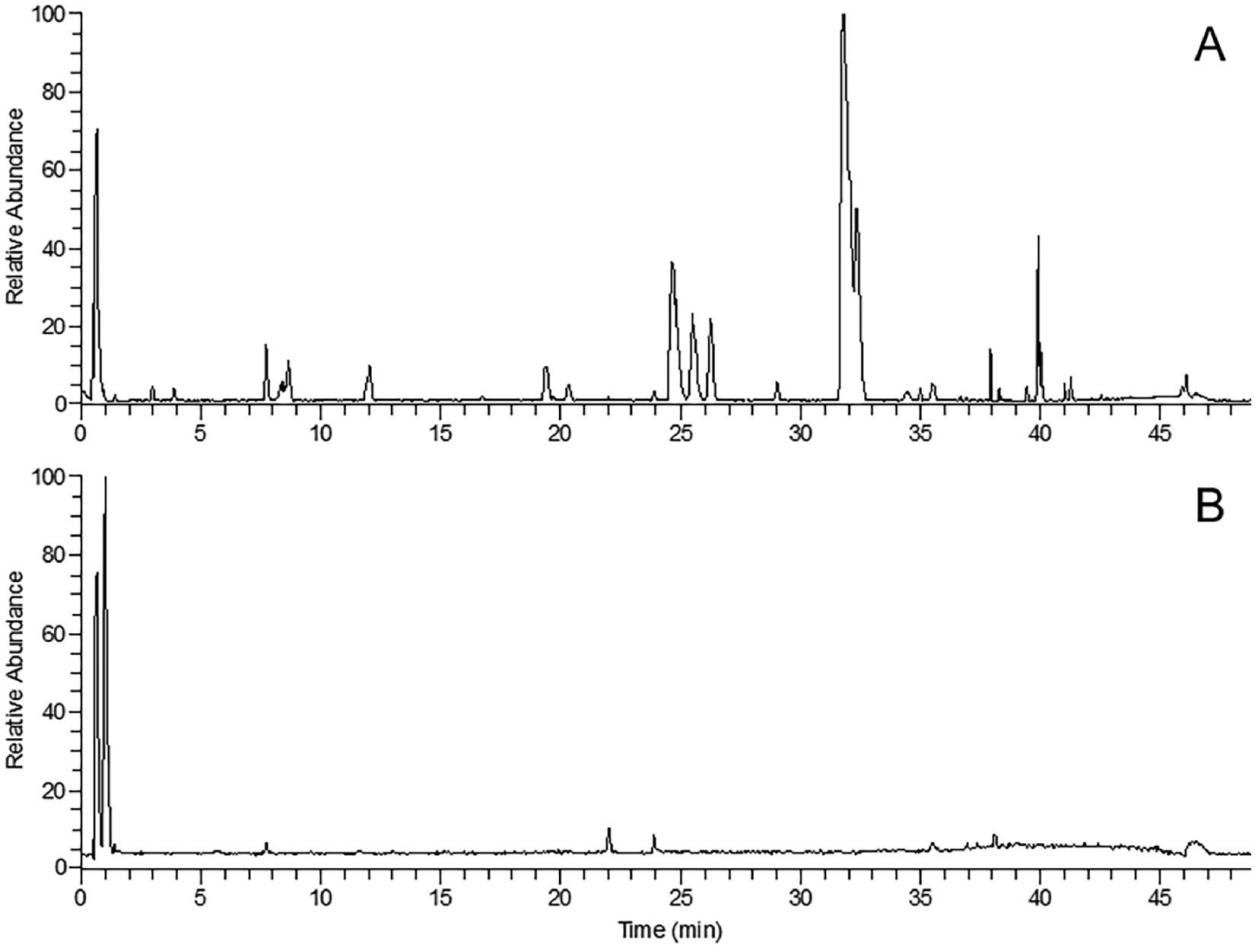
The TIC chromatogram of UPLC-MS in both positive mode (A) and negative mode (B) for TWMM.

The extract of TWMM was analyzed using UPLC Q-Orbitrap MS/MS. The total ion current (TIC) chromatograms obtained in both positive and negative modes are shown in Figure 1. The 90 components can be divided into 8 classes: 27 flavonoids, 17 iridoids, 16 alkaloids, 10 triterpenoids, 9 phenols, 9 organic acids, 1 phenylethanol, and 1 anthraquinone. Twenty-six components were identified by matching retention times, quasi-molecular ions, and MS/MS fragments with the standards. The other 64 components were identified by comparing their retention times and MS/MS fragments with those reported in the literature and databases. As shown in Tables 3 and 4, a total of 38 compounds were identified in positive ion mode and 52 compounds in negative ion mode. As an example of the identification process, compound no. 23 with detected ions at *m/z* 321.0996 and 292.0969 could be identified as berberine by comparison with the standard. The fragment at *m/z* 321.0996 was generated by the loss of CH_3_, and *m/z* 292.0969 was obtained by further loss of HCO.

**Table 3.** Compounds in TWMM detected by LC/MS in positive ion mode.

No.	RT (min)	Formula	[M + H]^+^ (*m/z*)	Identification	Mass error (ppm)	MS^2^ fragments
1*	0.64	C_7_H_14_O_2_N	144.1019	Stachydrine	0.588	144.1020,102.5555,98.0973,84.0814,72.0814,58.0660
2	0.68	C_6_H_12_O_2_N	130.0862	*L* (-)-pipecolinic acid	–0.424	84.0814,67.0550,56.0503
3*	8.62	C_20_H_24_O_4_N	342.1697	Magnoflorine	0.454	342.1701,297.1123,282.0889,265.0861,255.1029, 237.0910,219.0806
4	11.92	C_14_H_22_O_5_N_3_	312.1569	Leonurine	4.686	312.1569,181.0495,132.1133,114.1029,97.0765,72.0815
5	15.50	C_21_H_26_O_4_N	356.1859	Tetrahydropalmatine	0.773	356.1859,339.1075,204.1021,165.0914,151.0751
6	16.43	C_15_H_13_O_4_	257.0806	Liquiritigenin	–0.916	147.0440,137.0234,119.0494
7	16.80	C_21_H_19_O_11_	447.0921	Astragalin	–0.196	285.0758,253.0492,137.0235,91.0576
8*	16.80	C_22_H_23_O_10_	447.1302	Calycosin-7-*O*-*β*-*D*-glucoside	3.638	285.0758,270.0524,253.0492,225.0559,137.0235,91.0576
9	16.94	C_20_H_20_O_5_N	354.1334	Protopine	–0.591	354.1334,339.1098,324.0861,310.1075
10	18.69	C_19_H_18_O_4_N	324.1230	Stepharanine isomer	–0.045	324.1230,308.0879,294.0760,280.0950,266.0808
11	18.86	C_28_H_35_O_15_	611.1995	Hesperidin	4.014	355.0691,239.0948,129.0551
12	19.10	C_19_H_18_O_4_N	324.1236	Stepharanine isomer	1.652	324.1236,308.0895,294.0761,280.0962,266.0808
13*	19.78	C_19_H_18_O_4_N	324.1229	Stepharanine	–0.353	324.1229,308.0916,294.0762,280.0969,266.0806
14	19.89	C_15_H_11_O_6_	287.0549	Kaempferol	–0.503	287.0549,153.0182,84.9604
15	21.23	C_19_H_18_O_4_N	324.1226	Stepharanine isomer	–1.279	324.1223,308.0914,294.0761,280.0964,266.0792
16	24.63	C_19_H_14_O_4_N	320.0918	Coptisine	0.205	320.0918,318.0763,292.0968,277.0735,262.0863,249.0792
17	25.45	C_22_H_28_O_4_N	370.2014	Corydaline	0.176	370.2014,206.1178,165.0548
18*	25.48	C_20_H_20_O_4_N	338.1385	Jatrorrhizine	–1.019	338.1383,323.1143,308.0917,294.1123,279.0882
19*	25.65	C_20_H_18_O_4_N	336.1231	Epiberberine	0.135	336.1231,320.0918,308.0914,294.1123,279.0893
20*	26.26	C_20_H_20_O_4_N	338.1385	Columbamine	–0.487	338.1385,323.1143,308.0916,294.1124,279.0887
21*	28.45	C_22_H_23_O_9_	431.1335	Ononin	–0.368	269.0809,254.0572,253.0503,237.0552,213.0910
22	29.68	C_21_H_20_O_4_N	350.1389	Dehydrocavidine	0.472	350.1389,335.1131,334.1075,306.1124
23	30.50	C_15_H_11_O_4_	255.0653	Daidzein	0.489	255.0653,237.0546,227.0707
24*	31.82	C_20_H_18_O_4_N	336.1232	Berbine	0.403	336.1232,320.0918,306.0760,292.0969,278.0814
25*	32.33	C_21_H_22_O_4_N	352.1542	Palmatine	0.924	352.1547,336.1232,322.1075,308.1283,294.1129
26*	32.40	C_19_H_16_O_4_N	322.1072	Berberrubine	–0.573	294.1126,278.0811,102.9706,84.9604,74.9316
27*	34.40	C_16_H_13_O_5_	285.0750	Calycosin	–0.246	285.0757,270.0522,253.0496,225.0546, 214.0621,197.0599,137.0234
28	35.22	C_22_H_24_O_4_N	366.1699	Dehydrocorydaline	–0.259	366.1699,350.1387,336.1228
29	35.52	C_21_H_20_O_4_N	350.1389	Dehydrocavidine isomer	0.587	350.1389,334.1076,306.1125
30	35.59	C_28_H_33_O_14_	593.1863	Linarin	–0.307	447.1289,285.0760,129.0548,85.0290
31	38.04	C_15_H_11_O_5_	271.0602	Baicalein	0.185	271.0602,208.9540,146.9613,69.0706
32	38.61	C_16_H_13_O_6_	301.0709	Chrysoriol	0.749	286.0471,258.0525,229.0499,213.0543
33*	39.39	C_16_H_13_O_4_	269.0810	Formononetin	0.463	269.0810,254.0569,237.0547,226.0625,213.0911, 197.0599,137.0235,118.0416,107.0496
34	39.51	C_16_H_29_O_2_	253.2160	Hexadecadienoic acid	–0.816	109.1015,95.0861
35	40.76	C_21_H_23_O_8_	403.1406	Nobiletin	4.678	403.1406,388.1141,373.0919,327.0851
36	41.43	C_30_H_47_O_3_	455.3535	3-oxo-olean-12-en-28-oic acid/3-keto-oleanolic acid	3.246	455.3535,437.3405,229.1949,201.1633,191.1785,1 89.1638,159.1168,133.1013,109.1016,95.0861
37*	44.37	C_30_H_47_O_4_	471.3473	2α-hydroxyoleanic acid	0.856	471.3473,425.3422,235.1693,189.1639
						
38	45.42	C_30_H_49_O_3_	457.3671	Ursolic acid/oleanolic acid	–1.141	411.3626,203.1795,201.1645,189.1642,175.1489, 161.1327,149.1324,147.1172,135.1171,121.1014

*Compounds identified by comparison with reference standards.

**Table 4.** Compounds in TWMM detected by LC/MS in negative ion mode.

No.	RT (min)	Formula	[M − H]^−^ (*m/z*)	Identification	Mass error (ppm)	MS^2^ fragments
39	0.89	C_4_H_5_O_5_	133.0131	Malic acid	–0.374	133.0131,71.0125
40	1.94	C_8_H_7_O_4_	167.0343	Vanillic acid	2.483	123.0440,81.0330
41	2.29	C_16_H_23_O_10_	375.1302	Loganic acid or isomer	4.336	113.0233,101.0233,85.0283,71.0125,59.0126,343.1819,304.3728, 280.5988,186.9071
42*	2.46	C_8_H_9_O_3_	153.0550	Hydroxytyrosol	2.478	126.9022,123.0440,122.0362,108.0204,96.9588,95.0492
43	2.70	C_16_H_23_O_10_	375.1302	Loganic acid or isomer	1.627	151.0749,113.0229,101.0232,85.0279,71.0125,59.0126
44	2.87	C_16_H_17_O_9_	353.0887	Neochlorogenic acid	5.640	191.0554,179.0342,173.0451,161.0233,155.0337, 135.0440,133.0282
45*	2.94	C_14_H_19_O_7_	299.1139	Salidroside	4.582	113.0232,101.0232,89.0231,162.8383, 126.8801,119.0491,71.0125,59.0125
46	3.38	C_21_H_27_O_13_	487.1470	Cistanoside F	4.891	245.0459,203.0345,179.0342,161.0234,135.0440,113.0231
47	3.56	C_10_H_13_O_5_	213.0764	Nuzhenal A or isomer	3.051	215.0094,171.0195,144.0080,122.8930,61.9870,59.0125
48	3.76	C_7_H_5_O_3_	137.0241	Protocatechualdehyde	5.324	137.0240,119.0112,93.0333,136.0155,108.0204,66.0366, 61.9870
49	5.27	C_16_H_17_O_9_	353.0887	Chlorogenic acid	5.640	191.0554,179.0342,161.0233,135.0436,127.0389,102.9473, 85.0282
50	5.72	C_17_H_19_O_9_	367.1036	3-*O*-Feruloylquinic acid	3.382	193.0500,134.0362
51	6.06	C_16_H_17_O_9_	353.0887	Cryptochlorogenic acid	5.640	191.0555,179.0342,173.0448,161.0237,155.0339, 135.0440,111.0440,93.0332
52	6.17	C_9_H_7_O_4_	179.0343	Caffeic acid	2.317	143.8641,141.8670,135.0441,134.0361, 107.0489,103.9190,99.9245,90.9232
53	9.12	C_16_H_23_O_10_	375.1302	Loganic acid or isomer	1.627	85.0282,59.0123
54	10.36	C_9_H_7_O_3_	163.0393	Coumaric acid	2.020	119.0491,108.9363,93.0333
55	15.38	C_21_H_19_O_12_	463.0899	Isoquercetin	5.976	463.0899,301.0351,300.0275
56	15.75	C_35_H_45_O_20_	785.2507	Echinacoside	1.019	785.2507,179.0344,161.0235,623.6187,398.2031,244.7829
57	16.65	C_25_H_31_O_14_	555.1724	10-Hydroxyoleuropein/ligustalosideA	2.824	223.0593,151.0391,101.0229,89.0230,538.7717,431.5094, 367.6147,330.2907,274.4666,59.0124
58	18.33	C_25_H_29_O_15_	569.1519	Oleuropeinic acid	3.169	363.1089,331.0827,221.0084,209.0451, 177.0184,151.0391,133.0286,123.0440,195.0293
59	19.02	C_27_H_29_O_16_	609.1462	Rutin	2.017	609.1462,301.0349,300.0276,394.4090, 343.0453,302.0385,178.9981
60	19.53	C_21_H_19_O_12_	463.0887	Hyperoside	3.450	300.0277,271.0255,255.0313,178.9990,151.0028
61	19.57	C_31_H_41_O_18_	701.2300	Neonuzhenide	1.767	539.1758,469.1359,135.0440,101.0230, 701.2300,437.1450,315.1085
62*	19.94	C_21_H_19_O_11_	447.0939	Luteolin-7-*O*-glucoside	3.830	447.0939,285.0405,327.0508,286.0441,284.0328
63	20.28	C_34_H_43_O_19_	755.2415	Forsythoside B	1.224	593.2094,179.0337,161.0233
64*	21.99	C_29_H_35_O_15_	623.1981	Verbascoside	1.626	623.1981,461.1670,315.1082,179.0338, 161.0236,135.0441,113.0232
65	23.26	C_25_H_29_O_14_	553.1572	Ligustrosidic acid	3.648	347.1141,329.1022,315.0883,235.0255, 209.0450,195.0293,177.0186,151.0392,101.0230
66*	23.94	C_31_H_41_O_17_	685.2358	Specnuezhenide	2.881	523.1809,453.1404,421.1508,299.1136, 223.0606,181.0498,179.0555,121.0283,89.0231
67	24.07	C_27_H_29_O_14_	577.1570	Apigenin-7-*O*-rutinoside	3.115	577.1570,269.0457,516.5734,383.2473,311.0563, 270.0490,171.9616
68	24.69	C_21_H_19_O_10_	431.0985	Apigenin-7-*O*-glucoside	2.939	431.0985,269.0445,268.0378,311.0562,197.8080,160.8412
69*	29.45	C_25_H_31_O_13_	539.1780	Oleuropein	3.863	345.0996,327.0869,307.0824,275.0928, 223.0607,191.0342,153.0544, 149.0234,139.0389,119.0377
70	34.01	C_27_H_35_O_14_	583.2039	[M-H + HCOOH]-ligulucidumosideA	3.031	403.1255,223.0605,151.0390,123.0441, 101.0233,351.1075,319.0839,179.0547
71*	34.49	C_16_H_11_O_5_	283.0617	Physcion	5.653	240.0419,268.0377,239.0345,211.0395,184.0522,148.0156
72	34.66	C_31_H_39_O_15_	651.2278	Martynoside	–0.870	475.1821,193.0499,175.0393,160.0155
73	34.86	C_25_H_31_O_12_	523.1827	Ligustroside	3.244	291.0876,259.0979,223.0607,171.0294,139.0394,101.0232, 89.0229
74	35.10	C_15_H_9_O_6_	285.0408	Luteoline	4.931	285.0408,175.0393,151.0026,133.0280,107.0126,268.0375, 162.8384
75	35.68	C_33_H_43_O_18_	727.2466	Acetylnicotiflorine	3.038	495.1502,341.1252,299.1148,281.1036,223.0607,121.0280, 89.0229,611.7267,463.1580
76	36.36	C_25_H_27_O_12_	519.1516	6′-*O*-*trans*-Cinnamoyl-8-epikingisidicacidor isomer	3.655	189.0554,183.0653,165.0550,161.0559, 147.0441,121.0647,69.0332,59.0125
77*	36.90	C_48_H_63_O_27_	1071.3563	Oleonuezhenide	1.118	523.1824,453.1409,299.1137,223.0608,
78	37.14	C_30_H_25_O_13_	593.1309	Tiliroside	3.191	593.1309,447.0937,285.0403
79	37.34	C_48_H_63_O_27_	1071.3567	Oleonuezhenide or isomer	1.463	523.1823,453.1409,421.1511,299.1137,223.0610
80	38.12	C_15_H_9_O_5_	269.0459	Apigenin	5.390	269.0459,201.0550,183.0446,151.0027,149.0234, 117.0333,107.0125
81*	39.33	C_42_H_69_O_16_	829.4594	Astragaloside IV	1.613	829.4594,783.4568
82	39.70	C_10_H_13_O_5_	213.0764	Nuzhenal A or isomer	3.051	215.0093,122.8930,171.0191,144.0081,61.9871
83*	39.87	C_44_H_71_O_17_	871.4654	Astragaloside II	–3.657	871.4654,825.4614
84	40.14	C_48_H_77_O_18_	941.5126	Soyasaponin I	2.324	941.5126,705.7660,116.9273
85*	40.48	C_44_H_71_O_17_	871.4692	Isoastragaloside II	0.692	871.4692,825.4577
86*	41.48	C_46_H_73_O_18_	913.4818	Astragaloside I	2.921	913.4818,867.4780
87	41.79	C_46_H_73_O_18_	913.4813	IsoastragalosideI	2.319	913.4813,867.4747
88	41.92	C_30_H_47_O_5_	487.3441	Tormentic acid	4.635	487.3441,488.3474,470.3380,469.3336,425.3817, 394.9604,324.6631,274.6215,113.2870
89	44.28	C_39_H_53_O_7_	633.3798	3-*O*-*cis-p*-Coumaroyltormentic acid/3-*O*-*trans-p*-Coumaroyltormentic acid	1.862	633.3798,145.0280,580.6879,461.3018, 365.2326,162.8382,116.9270
90	46.08	C_39_H_53_O_6_	617.3853	3*β*-*O*-*trans-p*-Coumaroylmaslinicacidor isomer/3*β*-*O*-*cis-p*-Coumaroylmaslinicacidor isomer	1.886	617.3848,145.0286,412.5469,315.0490,303.2346,241.0108

*Compounds identified by comparison with reference standards.

#### GC-MS/MS-based screening and identification

**Figure 2.**
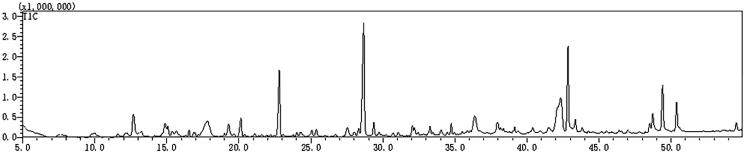
The TIC chromatogram of GC-MS for TWMM.

The volatile components of TWMM were detected by GC-MS 2010, and 45 compounds were identified using the NIST database. The GC-MS chromatogram of TIC traces is depicted in Figure 2 and the compounds identified from TWMM are listed in Table 5. Peak area normalization was applied to determine the relative content of each compound.

**Table 5.** Compounds in TWMM detected by GC/MS.

No.	RT (min)	Formula	Molecular weight (*m/z*)	Identification	SI	Retention index	Ratio (%)
91	9.77	C_6_H_8_O_3_	128.126	Glycidyl acrylate	85	865	0.24
92	11.57	C_3_H_6_O_2_	74.078	Acetol	91	698	0.41
93	12.17	C_9_H_17_NO_7_	251.234	Muramic acid	76	2221	0.52
94	12.68	C_5_H_6_O_2_	98.100	Furfuryl alcohol	80	885	2.78
95	13.22	C_7_H_14_O_2_	130.185	Methyl 2-ethylbutanoate	81	820	1.01
96	14.88	C_2_H_6_O_2_	62.068	Ethylene glycol	89	705	1.50
97	15.06	C_6_H_8_O_4_	144.125	2,4-Dihydroxy-2,5-dimethyl-3(2H)-furan-3-one	88	1173	0.82
98	15.36	C_5_H_6_O_2_	98.100	1,2-Cyclooctanedione	93	942	0.62
99	15.66	C_6_H_16_O_2_Si	148.275	Diethoxydimethylsilane	75	679	0.49
100	16.54	C_6_H_6_O_2_	110.111	5-Methyl furfural	96	920	0.47
101	16.85	C_5_H_4_O_3_	112.083	Citraconic anhydride	90	1068	0.31
102	16.96	C_4_H_6_O_2_	86.090	Gamma-Butyrolactone	83	825	0.18
103	17.84	C_3_H_8_O_3_	92.094	Glycerol	80	967	1.68
104	19.30	C_6_H_8_O_3_	128.126	4-Hydroxy-2,5-dimethylfuran-3(2H)-one	84	1022	1.48
105	20.14	C_6_H_6_O_3_	126.110	Maltol	84	1063	1.60
106	22.81	C_6_H_8_O_4_	144.125	4H-Pyran-4-one, 2,3-dihydro-3,5-dihydroxy-6-methyl	87	1269	5.21
107	23.76	C_5_H_10_O	86.132	Cyclopentanol	85	788	0.19
108	24.03	C_6_H_6_O_4_	142.109	3,5-Dihydroxy-2-methylpyran-4-one	88	1193	0.34
109	25.07	C_7_H_12_O_3_	144.168	Ethyl 2-methylacetoacetate	80	956	0.58
110	25.38	C_8_H_8_O	120.148	Coumaran	92	1036	0.74
111	28.31	C_5_H_10_O_4_	134.130	1,2,3-Propanetriol,1-acetate	88	1091	0.66
112	28.67	C_6_H_6_O_3_	126.110	5-Hydroxymethylfurfural	90	1163	10.67
113	29.36	C_9_H_10_O_2_	150.174	1-(4-Hydroxy-3-methylphenyl) ethanone	89	1363	0.98
114	32.19	C_8_H_10_O_3_	154.163	Syringol	87	1279	0.53
115	32.44	C_6_H_12_O_5_	164.156	1,4-Anhydro-D-sorbitol	82	1530	0.34
116	33.28	C_9_H_8_O_2_	148.159	Cinnamic acid	94	1357	0.70
117	33.48	C_14_H_22_O	206.324	3,5-Di-*tert*-Butylphenol	88	1555	0.25
118	34.45	C_6_H_9_NO_3_	143.141	Methyl DL-pyroglutamate	87	1091	0.25
119	34.75	C_8_H_10_O_2_	138.160	4-Hydroxyphenyl ethanol	94	1356	0.78
120	34.97	C_13_H_28_O	200.361	1-Hexoxy-5-methylhexane	85	1325	0.25
121	35.87	C_6_H_6_N_2_O	122.125	Nicotinamide	94	1197	0.50
122	36.38	C_4_H_9_NO_5_	151.118	2-(Hydroxymethyl)-2-nitropropan-1,3-diol	78	1444	1.80
123	37.97	C_6_H_10_O_5_	162.141	Levoglucosan	89	1404	1.49
124	38.18	C_8_H_8_O_4_	168.147	3-Hydroxy-4-methoxybenzoic acid	83	1560	0.64
125	39.14	C_12_H_20_O_7_	276.283	Triethyl citrate	88	1808	0.47
126	40.41	C_7_H_14_O_6_	194.180	Methyl-*β*-D-glucopyranoside	80	1714	0.81
127	42.16	C_11_H_22_O_2_	186.291	3-Methyldecanoic acid	69	1407	3.24
128	42.34	C_7_H_14_O_6_	194.182	Methyl *α*-D-mannofuranoside	78	1667	5.56
129	42.85	C_38_H_68_O_8_	652.942	l-Ascorbyl dipalmitate	91	4765	6.59
130	43.36	C_12_H_15_NO_3_	221.252	Methyl *N*-acetyl-L-phenylalaninate	82	1794	1.25
131	48.53	C_18_H_36_O_2_	284.477	Stearic acid	88	2167	0.73
132	48.73	C_18_H_34_O_2_	282.461	Oleic acid	89	2175	1.88
133	48.73	C_18_H_34_O_2_	282.461	Elaidic Acid	89	2175	1.02
134	49.42	C_18_H_32_O_2_	280.445	Linoleic acid	93	2183	3.73
135	50.39	C_18_H_30_O_2_	278.430	*α*-Linolenic acid	95	2191	2.16

### Network analysis

#### Screening of active ingredients and targets

The 135 identified components were used as the foundation of network pharmacology. To acquire the formula-disease-related genes, the identified compounds were added to the TCMSP database to screen active ingredients and related targets. Following this, a total of 68 compounds and 287 targets were retrieved. Further, 646 genes related to DR were acquired from the CTD, OMIM, and DrugBank databases. Subsequently, after the intersection, 57 shared targets were obtained.

#### Obtaining key targets

**Figure 3.**
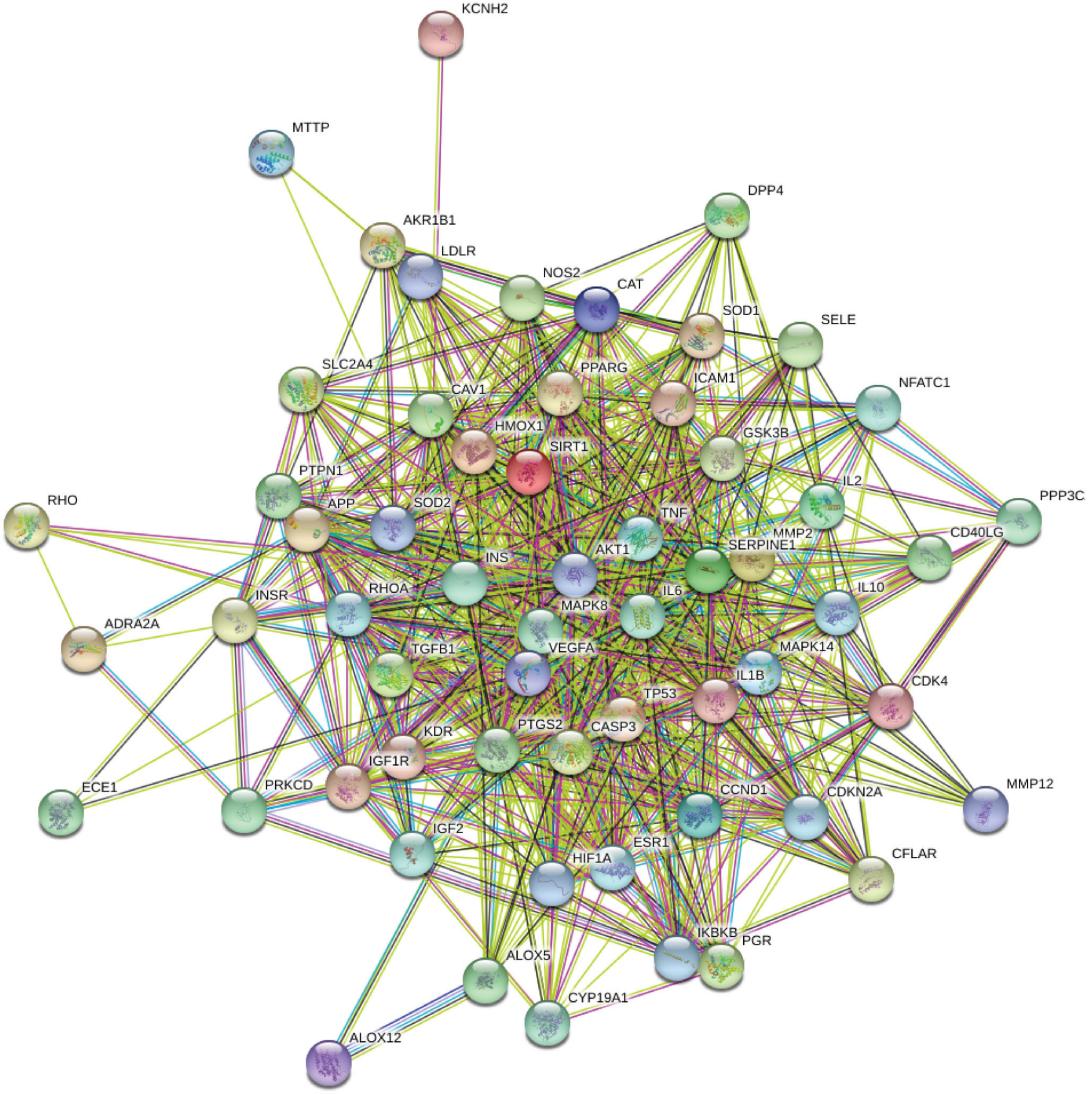
The PPI network of 57 intersection gene targets (colour indicates query proteins and first shell of interactors; filled nodes indicate some 3D structure is known or predicted; line thickness indicates the strength of data support).

**Figure 4.**
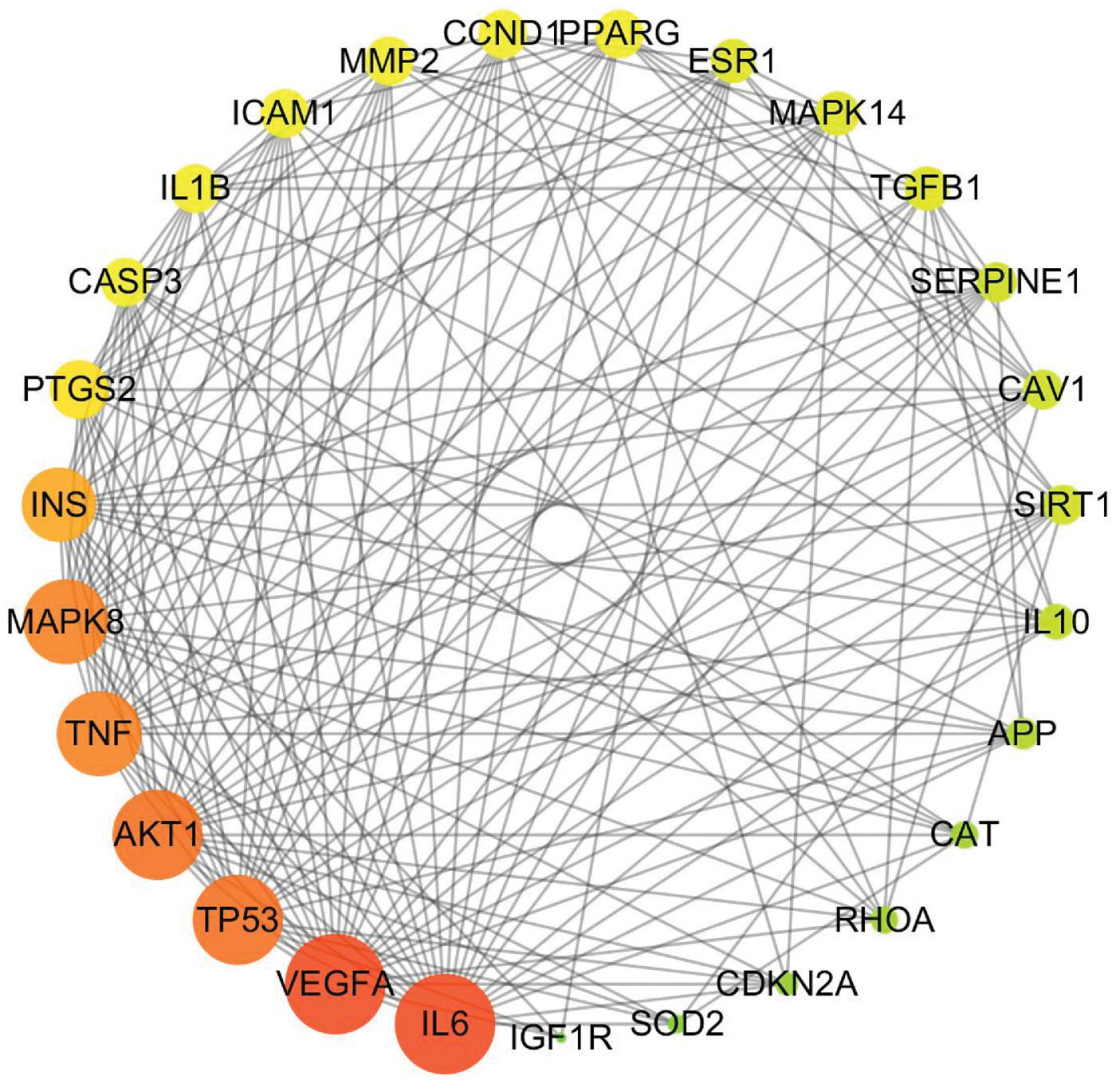
The 27 predicted key targets of TWMM in the therapy of DR (point size represents the degree value; colour from red to green represents the correlation from high to low).

The STRING database was used to form the intersection gene targets into a complex protein-protein network. The core gene targets are shown in Figure 3. The minimum confidence was set at 0.4 and Cytoscape 3.5.4 was used for further screening using the following node criteria: betweenness centrality ≥ 0.04, closeness centrality ≥ 0.6, and degree ≥ 27. Consequently, a total of 27 proteins were acquired as key candidate targets of DR. The 27 key targets are shown in Figure 4 and are listed in Table 6.

**Table 6.** The core targets of TWMM in treating diabetic retinopathy.

Gene official symbol	UniProtKB	Gene official symbol	UniProtKB	Gene official symbol	UniProtKB
IL6	P05231	IL1B	P01584	CAV1	Q03135
VEGFA	P15692	ICAM1	P05362	SIRT1	Q96EB6
TP53	P04637	MMP2	P08253	IL10	P22301
AKT1	P31749	CCND1	P24385	APP	P05067
TNF	P01375	PPARG	P37231	CAT	P04040
MAPK8	P45983	ESR1	P03372	RHOA	P61586
INS	P01308	MAPK14	Q16539	CDKN2A	P42771
PTGS2	P35354	TGFB1	P01137	SOD2	P04179
CASP3	P42574	SERPINE1	P05121	IGF1R	P08069

#### Core analysis and map of diseases & functions

**Figure 5.**
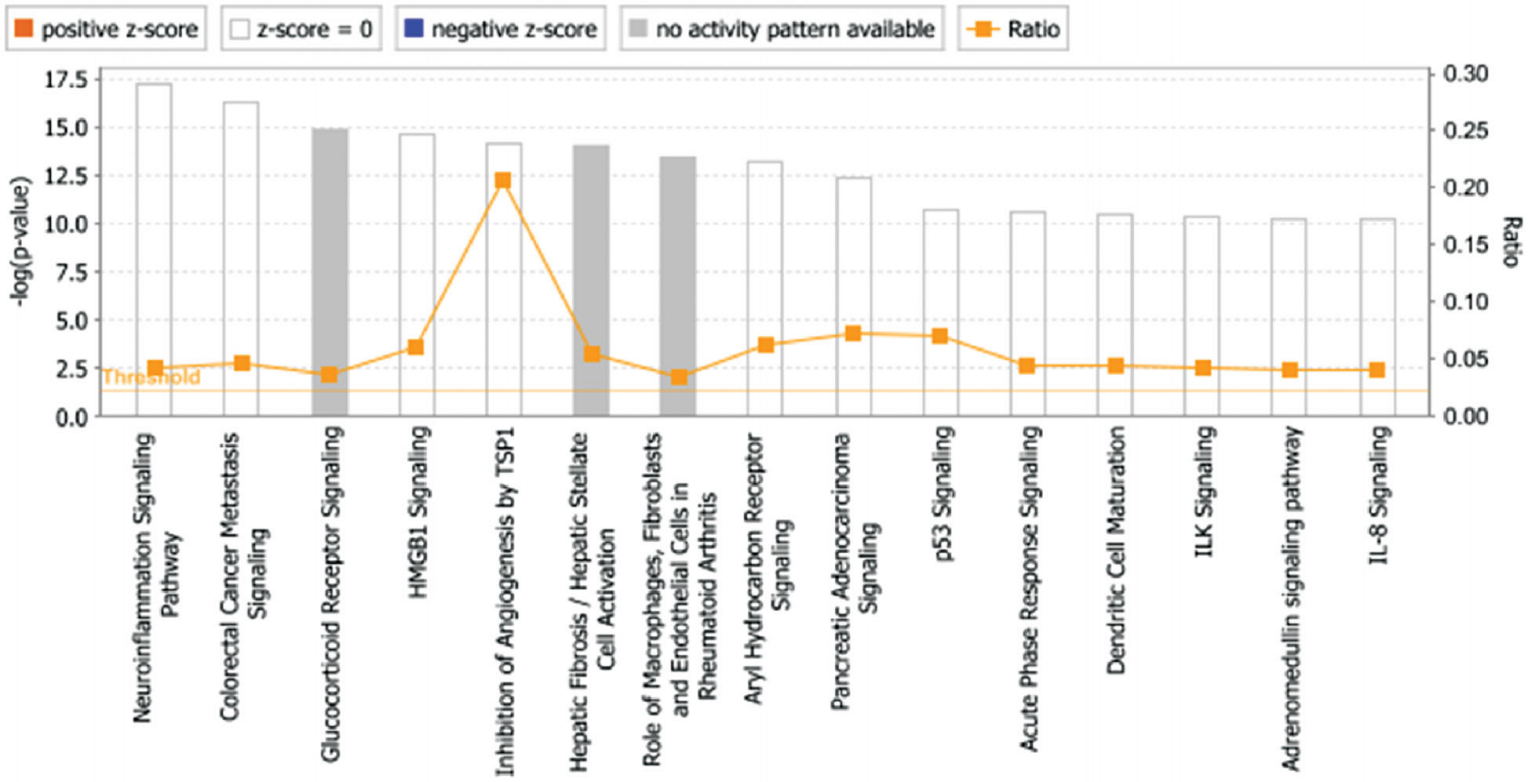
The core analysis of early DR treated with TWMM (–Log *p*-value represents the pathway correlation; ratio represents the ratio of the number of molecules to the total number of molecules in the pathway).

The 27 key targets are analyzed by the "Core Analysis" function in IPA. As shown in Figure 5, the top ten signalling pathways associated with TWMM and DR are listed: (1) Neuroinflammation Signalling Pathway; (2) Coloractal Cancer Metastasis Signalling; (3) Glucocorticoid Receptor Signalling; (4) HMGB1 Signalling; (5) Inhibition of Angiogenesis by TSP1; (6) Hepatic Fibrosis/Hepatic Stellate Cell Activation; (7) Role of Macrophages, Fibroblasts and Endothelial Cells in Rheumatoid Arthritis; (8) Aryl Hydrocarbon Receptor Signalling; (9) Pancreatic Adenocarcinoma Signalling (pancreatic cancer signalling); (10) P53 Signalling. Due to the large number of pathways involved, the disease pathways were screened based on the number of targets involved and their correlation with DR, among which the neuroinflammatory pathway, the inhibitory effect of TSP1 on angiogenesis, and the glucocorticoid receptor signalling pathway were of high relevance.

**Figure 6.**
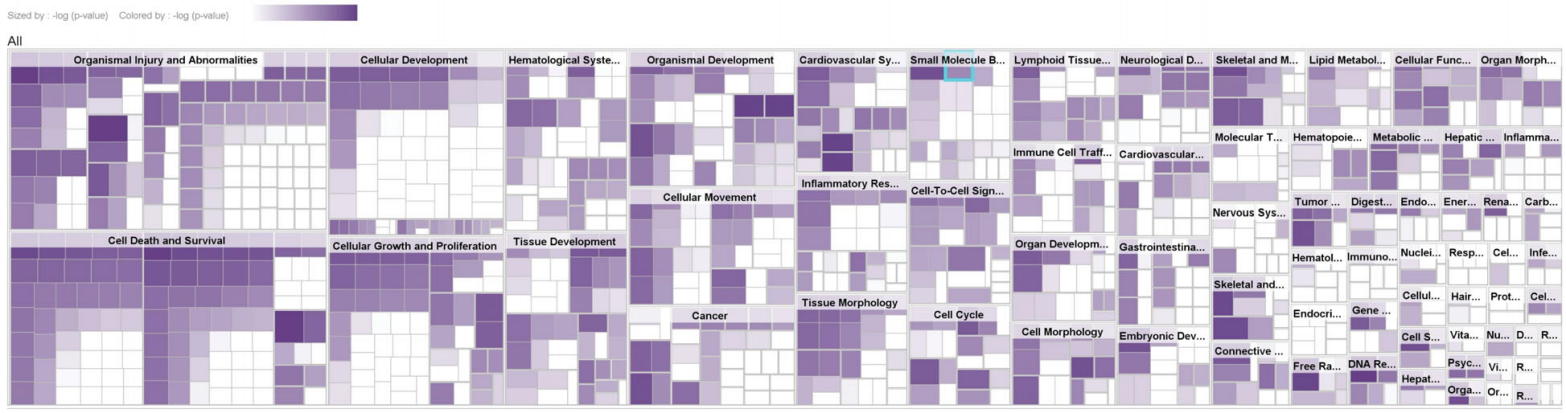
The map of diseases & functions (darker colour indicates smaller *p*-value).

A total of 72 functional entries related to DR were obtained by predicting the diseases and functions map of 27 core targets by IPA, namely organismal injury and abnormalities, cell death and survival, cellular development, cellular growth and proliferation, hematological system development, tissue development, organismal development, cellular movement, etc. As shown in Figure 6, it can be seen that the treatment of early DR by TWMM may be closely related to the apoptosis and proliferation of cells, the neogenesis of tissues and the process of the hematological system.

#### Construction of a compound-target-pathway network

**Figure 7.**
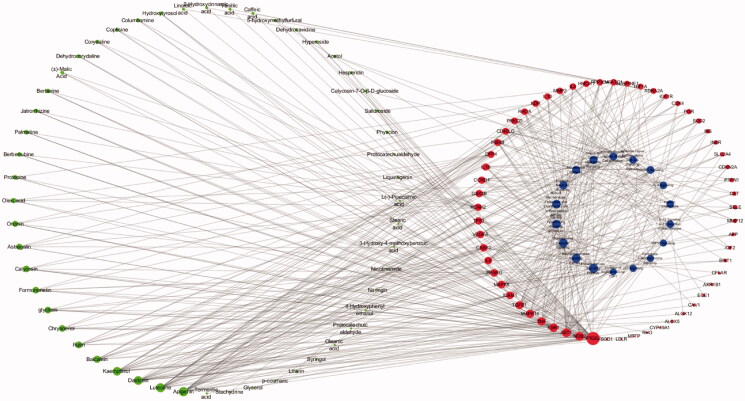
The compound-target-pathway network of TWMM in the treatment of DR (the left circle represents compounds; the outer circle of the right side represents the gene targets; the inner circle of the right side represents related pathways; point size from big to small represents the correlation from high to low).

As shown in Figure 7, a holistic compound-target-pathway network was framed by merging three networks to clarify the factors related to DR. The network is composed of 141 nodes and 477 edges. In the network analysis, the degree and betweenness centrality were selected to be the criteria indicators. The greater the value, the more critical the targets are. After sorting by degree and betweenness centrality, apigenin (degree: 22, betweenness centrality: 8.05E-02), luteolin (degree: 21, betweenness centrality: 5.99E-02), daidzein (degree: 19, betweenness centrality: 9.24E-02), kaempferol (degree: 18, betweenness centrality: 6.57E-02), baicalein (degree: 10, betweenness centrality: 2.81E-02), rutin (degree: 8, betweenness centrality: 2.35E-02), chrysoeriol (degree:7, betweenness centrality: 6.84E-03), glycitein (degree: 7, betweenness centrality: 8.52E-03) and formononetin (degree: 7, betweenness centrality: 1.27E-02) possess greater value of degree and betweenness. was suggested to play an important role in the activity of TWMM during the treatment of DR. Moreover, the top 6 targets sorted by degree value were PTGS2, NOS2, AKT1, ESR1, TNF, and MAPK14, which were inferred to be the most active targets. Based on the network and IPA analyses, the hepatic fibrosis signalling pathway, neuroinflammation signalling pathway, and glucocorticoid receptor signalling pathway were predicted as primary pathways involved in therapy.

Luteolin has been reported to inhibit proliferation and angiogenesis in human umbilical vein endothelial cells (HUVECs), human retinal microvascular endothelial cells (HRMECs), and retinal vascular endothelial rhesus (RF/6A) cells induced by VEGF (Bagli et al. 2004; Zhou et al. 2016). In addition, it was found to inhibit the advanced glycation end product (AGE)-forming of sorbitol and DPPH radical scavenging in rat lens (Hwang et al. 2019). Formononetin was reported to possess hypolipidemic, anti-inflammatory, and antioxidant activity, which can improve the clinical symptoms of DR. In addition, formononetin was shown to attenuate inflammatory responses by inhibiting the expression of interleukin (IL)-1β and intercellular adhesion molecule 1 (IcaM-1) at both protein and gene levels (Yang et al. 2005). Furthermore, treatment using formononetin for 16 weeks could ameliorate the clinical symptoms of hyperglycaemia and insulin resistance in diabetic animals. Moreover, oxidative stress burden was reduced by increased SIRT1 expression after oral administration of formononetin (Oza and Kulkarni 2019). Apigenin was reported to possess substantial anti-inflammatory and antioxidant activity via activation of nuclear factor erythroid 2-related factor 2 and haem oxygenase-1 (Li et al. 2020), while kaempferol was shown to reduce inflammatory and fibrogenic responses in NRK-52E cells induced by high glucose (Luo et al. 2021). The present results indicate that TWMM has multiple activities that can improve retinal microvascular function in DR.

### Bioanalytical method validation

**Figure 8.**
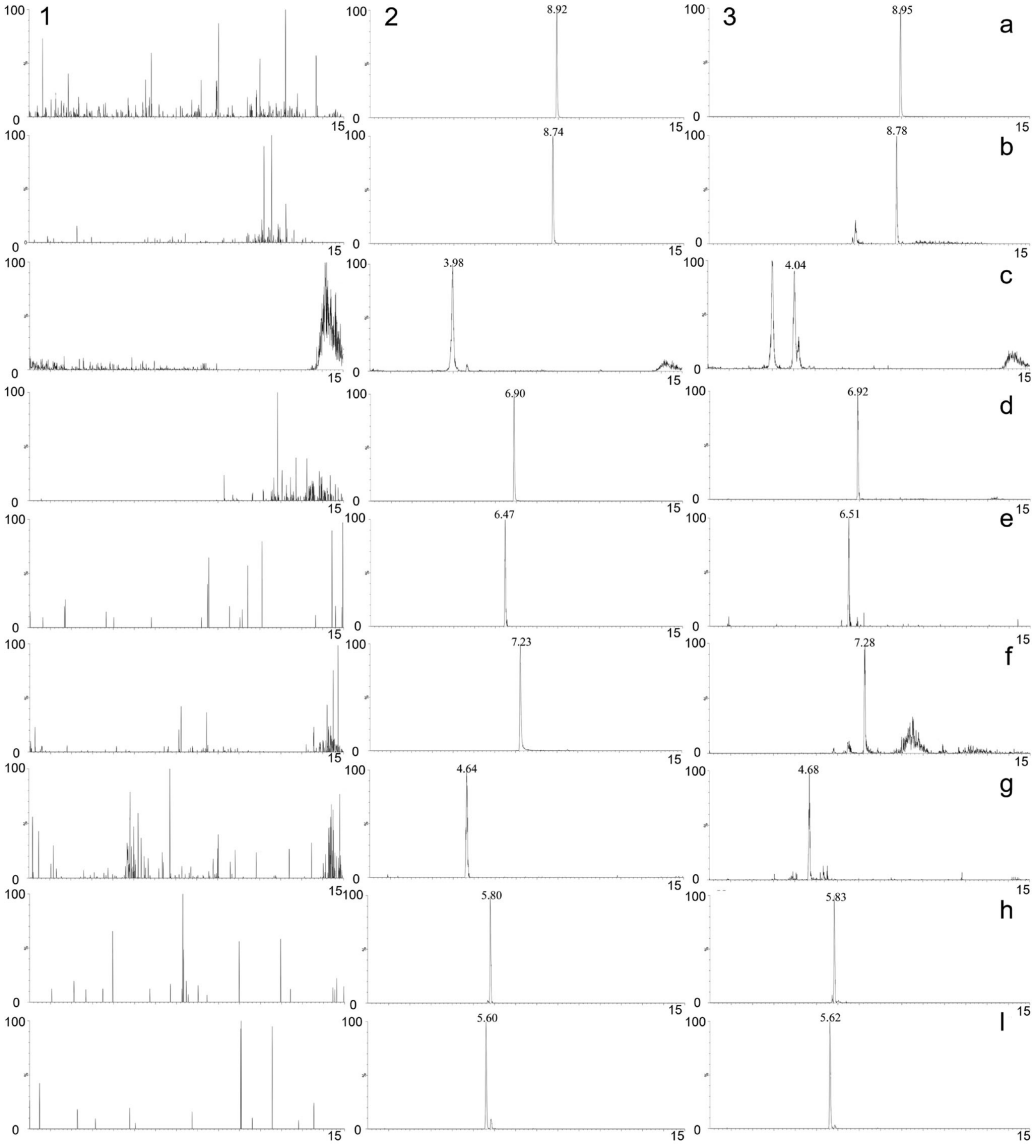
MRM chromatograms of tolbutamide (a), formononetin (b), chlorogenic acid (c), linarin (d), oleuropein (e), luteolin (f), jaspolyside (g), specnuezhenide (h) and verbascoside (i). (1) Blank plasma, (2) blank plasma spiked with the analytes and IS, (3) plasma sample after oral administration of TWMM.

The chromatograms of blank plasma, blank plasma spiked with the eight compounds and IS, and plasma after oral administration of TWMM are shown in Figure 8. The resultant chromatograms show that no significant peak interfered with the analysis.

The calibration curves of eight compounds were assessed by linear regression analysis with a weight factor of 1/*x^2^*. As shown in Table 7, all analytes had good linearity over the investigated range, and the correlation coefficient (*r*) of all calibration curves was greater than 0.9906.

**Table 7.** Calibration curves, correlation coefficients, linear ranges and LLOQ of 8 compounds.

Compounds	Calibration curve	*r*	Linear range (ng/mL)	LLOQ (ng/mL)
Oleuropein	*y* = 0.094*x*–0.021	0.9934	0.25–100	0.2
Chlorogenic acid	*y* = 0.064*x*–0.057	0.9906	0.5–200	0.3
Formononetin	*y* = 0.709*x*–0.180	0.9988	0.5–200	0.3
Verbascoside	*y* = 0.086*x*–0.092	0.9987	1–400	1.0
Linarin	*y* = 0.177*x*–0.058	0.9990	0.5–200	0.2
Luteolin	*y* = 1.031*x*–0.334	0.9971	0.5–200	0.2
Jaspolyside	*y* = 0.044*x*–0.024	0.9915	0.5–200	0.2
Specnuezhenide	*y* = 0.079*x*–0.038	0.9932	0.5–200	0.2

The intra- and inter-day precision and accuracy at each investigated concentration level are shown in Table 8. The intra- and inter-day accuracy (RE) ranged between −13.3% and 13.3%, while the precision (RSD%) was less than 13.5%. The above results corroborate the accuracy and precision of the method.

**Table 8.** Precision and accuracy of 8 compounds in rat plasma (*n* = 6).

Compounds	Spiked concentration (ng/mL)	Intra-day			Inter-day		
		Measured (ng/mL)	RE (%)	RSD (%)	Measured (ng/mL)	RE (%)	RSD (%)
Oleuropein	0.5	0.4 ± 0.1	–13.3	11.9	0.5 ± 0.1	–4.4	13.5
	5	4.9 ± 0.3	–3.0	5.2	5.1 ± 0.4	2.6	7.9
	100	100.8 ± 7.0	0.8	6.9	108.9 ± 10.1	8.9	9.3
Chlorogenic acid	1	1.1 ± 0.1	13.3	4.6	1.1 ± 0.1	12.8	5.9
	10	9.0 ± 0.5	–10.0	5.3	9.4 ± 0.6	–6.1	6.0
	200	193.2 ± 6.5	–3.4	3.4	209.6 ± 15.7	4.8	7.5
Formononetin	1	1.1 ± 0.1	11.7	8.8	1.1 ± 0.1	9.4	8.6
	10	10.3 ± 0.3	3.2	2.8	10.3 ± 0.4	3.2	4.1
	200	224.4 ± 6.5	12.2	2.9	221.8 ± 11.2	10.9	5.0
Verbascoside	2	2.0 ± 0.1	1.7	6.0	2.0 ± 0.2	2.2	7.4
	20	21.2 ± 1.3	5.8	6.2	21.6 ± 1.4	8.1	6.6
	400	424.0 ± 22.5	6.0	5.3	419.5 ± 16.4	4.9	3.9
Linarin	1	1.0 ± 0.1	3.3	10.0	1.1 ± 0.1	6.1	10.3
	10	10.2 ± 0.4	1.7	3.8	10.4 ± 0.3	3.8	3.2
	200	213.1 ± 4.1	6.5	1.9	217.3 ± 12.1	8.7	5.5
Luteolin	1	1.1 ± 0.1	11.7	8.8	1.1 ± 0.1	9.4	8.6
	10	10.0 ± 0.3	–0.5	3.0	10.3 ± 0.5	3.0	4.8
	200	221.7 ± 7.1	10.8	3.2	221.0 ± 8.9	10.5	4.0
Jaspolyside	1	1.1 ± 0.1	5.0	11.7	1.0 ± 0.1	2.8	11.0
	10	9.7 ± 1.0	–2.8	10.6	9.6 ± 0.8	–4.2	8.6
	200	200.3 ± 17.6	0.1	8.8	209.5 ± 16.5	4.8	7.9
Specnuezhenide	1	1.1 ± 0.1	10.0	10.0	1.1 ± 0.1	6.7	10.2
	10	10.3 ± 0.5	3.0	4.8	10.3 ± 0.5	3.1	5.2
	200	213.7 ± 7.6	6.9	3.6	215.0 ± 9.2	7.5	4.3

The extraction recoveries and matrix effects of QC samples are summarized in Table 9. The extraction recoveries of the eight analytes were between 85.5% and 113.3%, with RSD values less than 12.9%. The matrix effects of these analytes were within the acceptable range of 83.2–117.5%, with RSD values less than 15.4%. These results demonstrate that the obtained extraction recoveries and matrix effects were acceptable at different concentrations.

**Table 9.** Extraction recoveries and matrix effects of 8 compounds (*n* = 6).

Compounds	Spiked concentration (ng/mL)	Extraction recovery (%)	RSD (%)	Matrix effect (%)	RSD (%)
Oleuropein	0.5	95.8 ± 12.3	12.9	108.2 ± 13.1	12.1
	5	89.5 ± 6.0	6.7	89.6 ± 5.0	5.6
	100	98.4 ± 1.8	1.8	94.5 ± 2.3	2.5
Chlorogenic acid	1	86.7 ± 9.9	11.4	109.6 ± 9.9	9.0
	10	89.6 ± 9.5	10.6	93.4 ± 5.7	6.1
	200	113.3 ± 7.9	7.0	108.5 ± 7.9	7.3
Formononetin	1	97.2 ± 7.3	7.5	113.6 ± 4.9	4.3
	10	89.6 ± 2.0	2.3	86.6 ± 2.6	3.1
	200	90.1 ± 1.3	1.5	90.1 ± 0.5	0.6
Verbascoside	2	85.5 ± 6.2	7.3	117.5 ± 8.3	7.1
	20	94.1 ± 3.0	3.2	100.2 ± 4.9	4.9
	400	102.5 ± 3.4	3.4	95.4 ± 3.5	3.6
Linarin	1	101.8 ± 12.8	12.6	116.6 ± 11.2	9.6
	10	88.0 ± 4.3	4.9	86.8 ± 3.0	3.5
	200	96.1 ± 0.5	0.6	100.2 ± 0.7	0.6
Luteolin	1	101.5 ± 11.3	11.1	108.3 ± 7.8	7.2
	10	90.8 ± 7.2	7.9	95.7 ± 2.3	2.4
	200	91.1 ± 3.4	3.8	90.7 ± 4.5	5.0
Jaspolyside	1	101.1 ± 12.2	12.1	94.1 ± 14.5	15.4
	10	97.2 ± 3.8	3.9	83.2 ± 8.5	10.2
	200	95.9 ± 1.6	1.6	91.1 ± 2.0	2.2
Specnuezhenide	1	100.5 ± 4.6	4.6	117.0 ± 10.6	9.1
	10	88.2 ± 2.8	3.1	88.1 ± 2.4	2.7
	200	95.3 ± 1.8	1.9	93.5 ± 0.8	0.9

The stability tests were carried out under three freeze-thaw cycles, at 25 °C for 4 h, in an autosampler for 12 h, and at −80 °C for 7 d. The result shown in Table 10 demonstrates that the three analytes were stable under the investigated conditions.

**Table 10.** Stability of 8 compounds in rat plasma (*n* = 3).

Compounds	Concentration (ng/mL)	Room temperature (4 h, 25 °C)		Three freeze/ thaw cycles		Autosampler (12 h, 4 °C)		Long term (7 day, –80 °C)	
		Measured (ng/mL)	RSD (%)	Measured (ng/mL)	RSD (%)	Measured (ng/mL)	RSD (%)	Measured (ng/mL)	RSD (%)
Oleuropein	0.5	0.5 ± 0.1	10.8	0.5 ± 0.1	10.8	0.4 ± 0.1	13.3	1.0 ± 0.0	0.0
	5	4.3 ± 0.5	12.0	5.1 ± 0.2	3.0	0.5 ± 0.0	0.0	4.9 ± 0.3	5.9
	100	108.6 ± 7.8	7.2	103.6 ± 9.3	9.0	93.4 ± 8.6	9.2	113.9 ± 9.9	8.7
Chlorogenic acid	1	1.2 ± 0.1	4.9	1.1 ± 0.1	5.1	0.9 ± 0.1	6.2	1.0 ± 0.1	5.6
	10	9.2 ± 0.6	6.6	9.8 ± 0.8	7.6	9.1 ± 1.1	12.1	10.3 ± 0.5	4.8
	200	187.6 ± 4.2	2.3	212.6 ± 19.4	9.1	200.0 ± 14.0	7.0	214.4 ± 10.5	4.9
Formononetin	1	1.1 ± 0.1	10.2	1.0 ± 0.1	10.0	1.2 ± 0.1	4.9	1.1 ± 0.1	5.4
	10	11.8 ± 0.2	1.3	10.1 ± 1.0	0.6	10.1 ± 0.2	1.5	10.2 ± 0.2	2.0
	200	220.6 ± 3.5	1.6	206.3 ± 21.0	10.2	222.6 ± 3.6	1.6	216.0 ± 4.3	2.0
Verbascoside	2	2.2 ± 0.1	2.6	2.0 ± 0.1	2.8	2.1 ± 0.2	8.2	1.9 ± 0.2	7.9
	20	19.6 ± 1.0	5.3	21.8 ± 1.9	8.9	23.8 ± 1.8	7.7	21.3 ± 1.8	8.5
	400	427.6 ± 13.8	3.2	416.4 ± 17.6	4.2	402.4 ± 19.1	4.7	430.0 ± 6.2	1.4
Linarin	1	1.0 ± 0.1	11.2	1.0 ± 0.0	0.0	1.1 ± 0.1	9.1	1.1 ± 0.1	5.4
	10	11.9 ± 0.1	0.8	11.6 ± 0.5	4.1	10.2 ± 0.6	5.4	10.2 ± 0.4	3.5
	200	223.3 ± 5.0	2.2	219.4 ± 13.8	6.3	208.5 ± 2.0	1.0	221.0 ± 11.2	5.1
Luteolin	1	1.2 ± 0.1	4.9	1.1 ± 0.1	9.1	1.1 ± 0.1	5.1	1.0 ± 0.1	11.2
	10	11.3 ± 0.3	2.2	11.4 ± 0.8	7.3	10.0 ± 0.2	1.5	10.4 ± 0.6	6.2
	200	217.9 ± 11.7	5.4	218.0 ± 9.9	4.5	205.6 ± 0.9	0.4	221.9 ± 6.8	3.1
Jaspolyside	1	1.2 ± 0.1	4.9	1.1 ± 0.1	9.1	1.0 ± 0.1	10.0	1.0 ± 0.1	11.2
	10	11.1 ± 0.8	7.2	10.4 ± 0.4	2.9	11.0 ± 0.1	1.1	10.1 ± 0.2	1.7
	200	227.5 ± 9.8	4.3	206.0 ± 15.7	7.6	210.8 ± 2.6	1.2	216.0 ± 16.1	7.5
Specnuezhenide	1	1.0 ± 0.1	10.0	1.0 ± 0.0	11.9	1.1 ± 0.1	5.4	1.0 ± 0.1	5.4
	10	10.7 ± 0.2	1.7	11.5 ± 0.6	5.3	9.8 ± 0.6	6.2	10.3 ± 0.6	6.1
	200	212.7 ± 8.4	4.0	219.0 ± 8.4	3.8	198.2 ± 2.6	1.3	225.6 ± 9.0	4.0

### Application of the bioanalytical method

**Figure 9.**
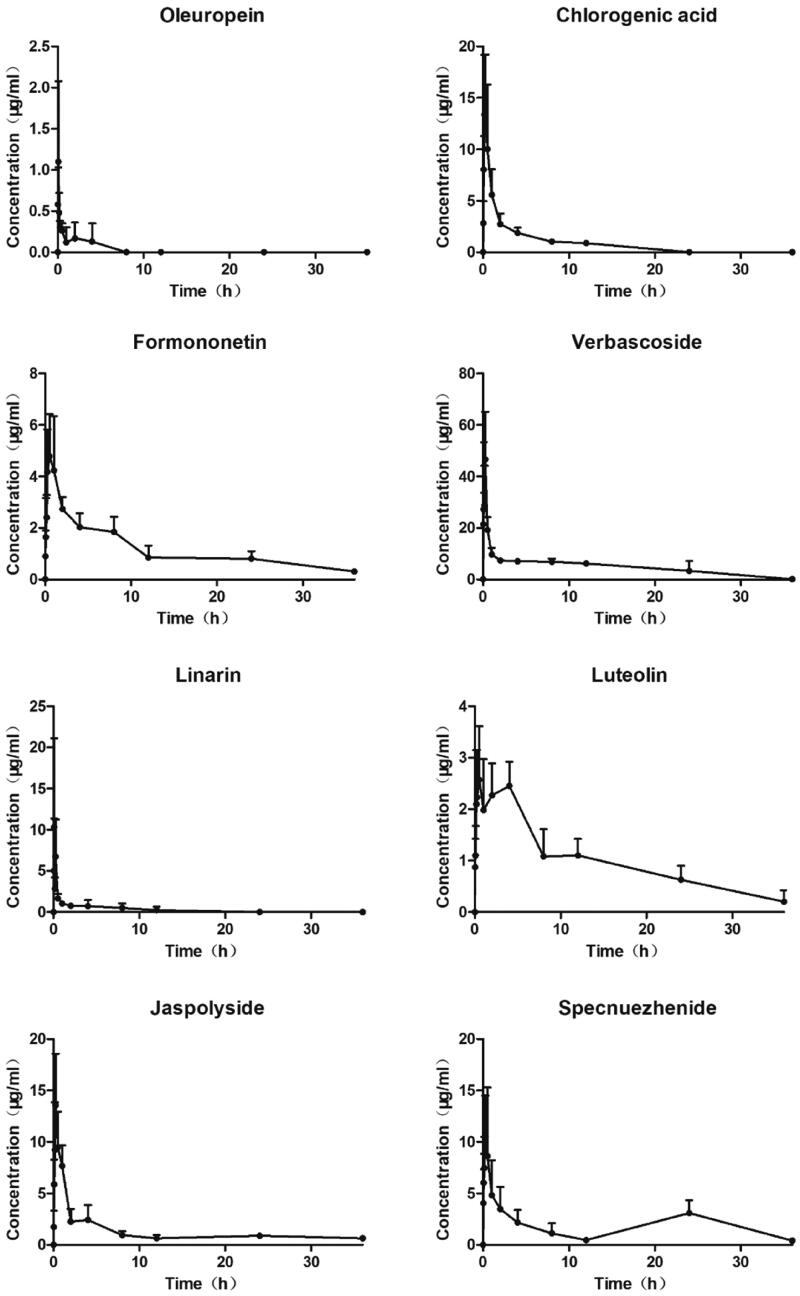
Plasma concentration–time curves of 8 compounds after oral administration of TWMM (*n* = 6, mean ± SD).

The plasma concentration-time curve after oral administration of TWMM is illustrated in Figure 9, and the determined pharmacokinetic parameters are shown in Table 11. According to DAS analysis, the *T*_max_ values of chlorogenic acid, formononetin, verbascoside, linarin, jaspolyside, and specnuezhenide were within 1 h, which suggests that these six compounds attained maximum plasma concentration rapidly. The *t*_1/2_ values show rapid elimination of oleuropein, chlorogenic acid, verbascoside, and linarin. The areas under the concentration-time curve (AUC_0–∞_) of oleuropein, chlorogenic acid, formononetin, verbascoside, linarin, luteolin, jaspolyside, and specnuezhenide were 1.03 ± 0.75, 33.95 ± 4.55, 45.96 ± 7.89, 174.81 ± 40.83, 12.04 ± 4.72, 43.98 ± 4.19, 53.37 ± 32.17, and 57.19 ± 30.25 mg/mL/min, respectively.

**Table 11.** Pharmacokinetic parameters of 8 compounds after oral administration of TWMM (*n* = 6).

Compounds	*T*_max_ (h)	*C*_max_ (ng/mL)	*t*_1/2_ (h)	AUC _(0–36)_ (h·ng/mL)	AUC _(0–∞)_ (h·ng/mL)	MRT _(0–36)_ (h)	MRT _(0–∞)_ (h)
Oleuropein	1.37 ± 1.60	0.90 ± 0.82	3.36 ± 0.00	1.03 ± 0.75	1.03 ± 0.75	1.53 ± 1.25	1.54 ± 1.25
Chlorogenic acid	0.39 ± 0.32	16.13 ± 4.17	2.71 ± 0.11	33.95 ± 4.55	33.95 ± 4.55	4.97 ± 0.71	4.97 ± 0.71
Formononetin	0.46 ± 0.29	6.17 ± 1.65	11.70 ± 2.09	40.78 ± 8.51	45.96 ± 7.89	10.69 ± 1.17	15.73 ± 1.90
Verbascoside	0.25 ± 0.14	45.47 ± 21.16	2.08 ± 0.38	174.81 ± 40.83	174.81 ± 40.83	8.90 ± 3.44	8.91 ± 3.44
Linarin	0.16 ± 0.10	12.78 ± 8.82	2.81 ± 0.21	12.04 ± 4.72	12.04 ± 4.72	4.26 ± 2.67	4.26 ± 2.68
Luteolin	1.71 ± 1.79	3.27 ± 0.78	11.14 ± 10.20	38.46 ± 7.61	43.98 ± 4.19	11.36 ± 1.23	18.20 ± 9.14
Jaspolyside	0.38 ± 0.31	14.25 ± 3.67	29.15 ± 12.65	46.59 ± 9.58	53.37 ± 32.17	11.42 ± 1.29	15.62 ± 10.83
Specnuezhenide	0.46 ± 0.29	13.33 ± 4.61	20.27 ± 25.00	48.62 ± 19.41	57.19 ± 30.25	11.02 ± 5.63	19.29 ± 16.60

### Validation of bioactivity

**Figure 10.**
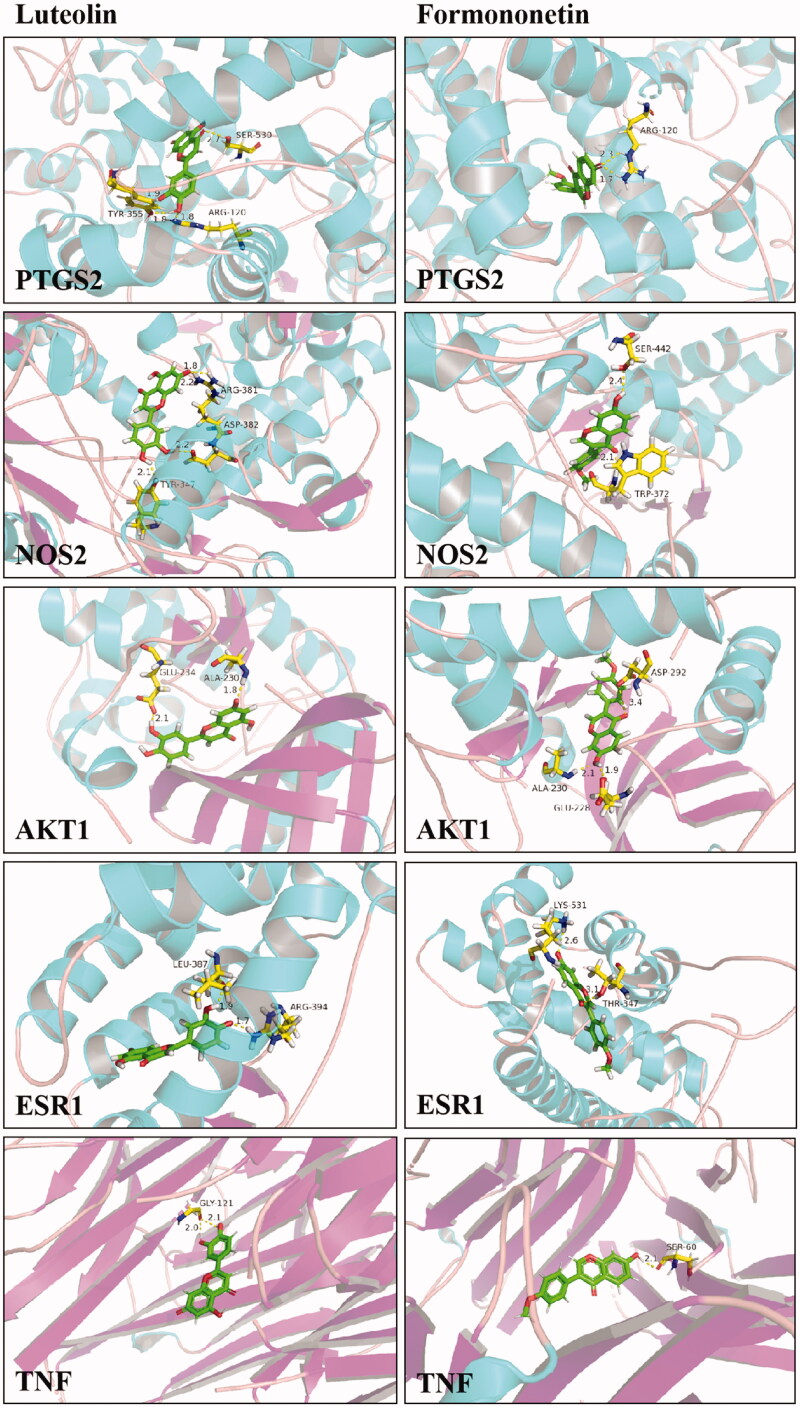
The 3D interaction diagrams of luteolin and formononetin.

The result of network pharmacology and identification of compounds *in vivo* revealed the potential ingredients and targets in the DR treatment of TWMM. To verified the bioactivity, the validated promoter or inhibitor binding sites of 5 targets were selected for docking with luteolin and formononetin. Then, PyMOL was used to verify and visualize the key hydrogen bonds of docking analysis (Figure 10). The -CDOCKER interaction energy ≤ −20 and hydrogen bonds indicated the good binding activity between two compounds and receptors. Furthermore, we used an assay of intracellular ROS and a luciferase reporter assay to verify the pharmacological activity of luteolin and formononetin. The results showed that luteolin and formononetin could alleviate the symptom of DR. More detail was added in Supplementary Materials.

### Application of network pharmacology in the pharmacokinetic study

Network pharmacology can be applied to screen compounds that have a high contribution in treating diseases, unveiling the target compounds to obtain better clinical adaptability that is similar to that of the parent herb or formulation. After systematic qualitative and network pharmacology analyses, apigenin, luteolin, daidzein, kaempferol, baicalein, rutin, chrysoeriol, glycitein, and formononetin were selected as potential target compounds based on degree and betweenness centrality. Furthermore, based on the qualitative analysis of plasma samples after oral administration of TWMM, luteolin and formononetin were found to immigrate into the blood. Therefore, luteolin and formononetin were selected as detectable *in vivo* markers having a high probability in the treatment of DR.

Network pharmacology can identify pharmacokinetic target compounds with high efficiency and accuracy. However, the pharmacokinetic parameters of the same compound in different herbs or prescriptions are not consistent. Wang et al. (2017) showed that the pharmacokinetic parameters of luteolin, in general, had a higher *T*_max_ and lower *t*_1/2_ than those of the extract. In addition, luteolin has been reported to have *T*_max_ and *t*_1/2_ values ranging between 0.50–2.83 h and 3.63–12.10 h in different herbal extracts and prescriptions, respectively (Guan et al. 2014; Cheruvu et al. 2018; Jia et al. 2020). Owing to the different *T*_max_ and *t*_1/2_ values from the various herbs and prescriptions, the influence on target compounds is diverse. Network pharmacology is adept at analyzing compound mixtures and presenting degrees to which each compound can be selected as a target molecule for pharmacokinetic studies of any herbal mixture.

Network pharmacology can also identify pharmacokinetic target compounds in herbs or prescriptions based on their clinical application. The different pathological conditions also influence the ADME of drugs, resulting in different therapeutic outcomes. On comparing the absorption rate of formononetin and luteolin in diabetic and healthy rats, Wei et al. (2017) observed that the absorption rate in the diabetic rats was lower than in the healthy ones, and the metabolism of formononetin in the former was more rapid, whereas that of luteolin was slower (Liu et al. 2021). Furthermore, ADME of the same compounds *in vivo* was greatly affected by the external environment. Therefore, these results prompt us to carry out a pharmacokinetic study using a DR mouse model in the future.

## Conclusions

Using network pharmacology, we focussed on the qualitative components of TWMM obtained using UPLC-MS and GC-MS to screen the key molecules that share the same targets with DR. Luteolin and formononetin were determined as the target compounds in explaining the pharmacokinetic properties of TWMM. This study provides a suitable combination strategy to unveil pharmacokinetic markers based on clinical application with high efficiency and clinical focus.

## Supplementary Material

Supplemental Material
