# Supplementary material for: Pharmacokinetic study of Tangwang Mingmu granule for the management of diabetic retinopathy based on network pharmacology
Source: Pharm Biol. 2021 Sep 30;59(1):1332–48. doi: 10.1080/13880209.2021.1979051 (PMC8491704; doi:10.1080/13880209.2021.1979051)
Supplement: Supplemental Material [file IPHB_A_1979051_SM0968.doc]

**Pharmacokinetic study of TangwangMingmu granules for the management of Diabetic Retinopathy based on network pharmacology**

Yucheng Wang, Beibei Xue, Xiaoli Wang, Qilong Wang, Erwei Liu*, Xiaopeng Chen*

*State Key Laboratory of Component-based Chinese Medicine, Tianjin University of Traditional Chinese Medicine, Tianjin 301617, China.*

^*^Corresponding authors:

Erwei Liu, No. 10, Poyang Lake Road, West Zone, Tuanbo New City, Jinghai District, Tianjin 301617, China.

E-mail addresses: liuwei628@hotmail.com

Xiaopeng Chen,No. 10, Poyang Lake Road, West Zone, Tuanbo New City, Jinghai District, Tianjin 301617, China.

E-mail addresses:[xpchen@tjutcm.edu.cn](mailto:xpchen@tjutcm.edu.cn)

**Figure S1. The 2D interaction diagrams of luteolin and formononetin with proteins**

**Figure S2. Effects of luteolin and formononetin on the ROS generation induced by HG (25mM/mL)**

**Figure S3. Dual‑luciferase reporter gene assay of luteolin and formononetin**

**Table S1. Comprehensive Interactions between the protein and luteolin**

**Table S2. Comprehensive Interactions between the protein and formononetin**


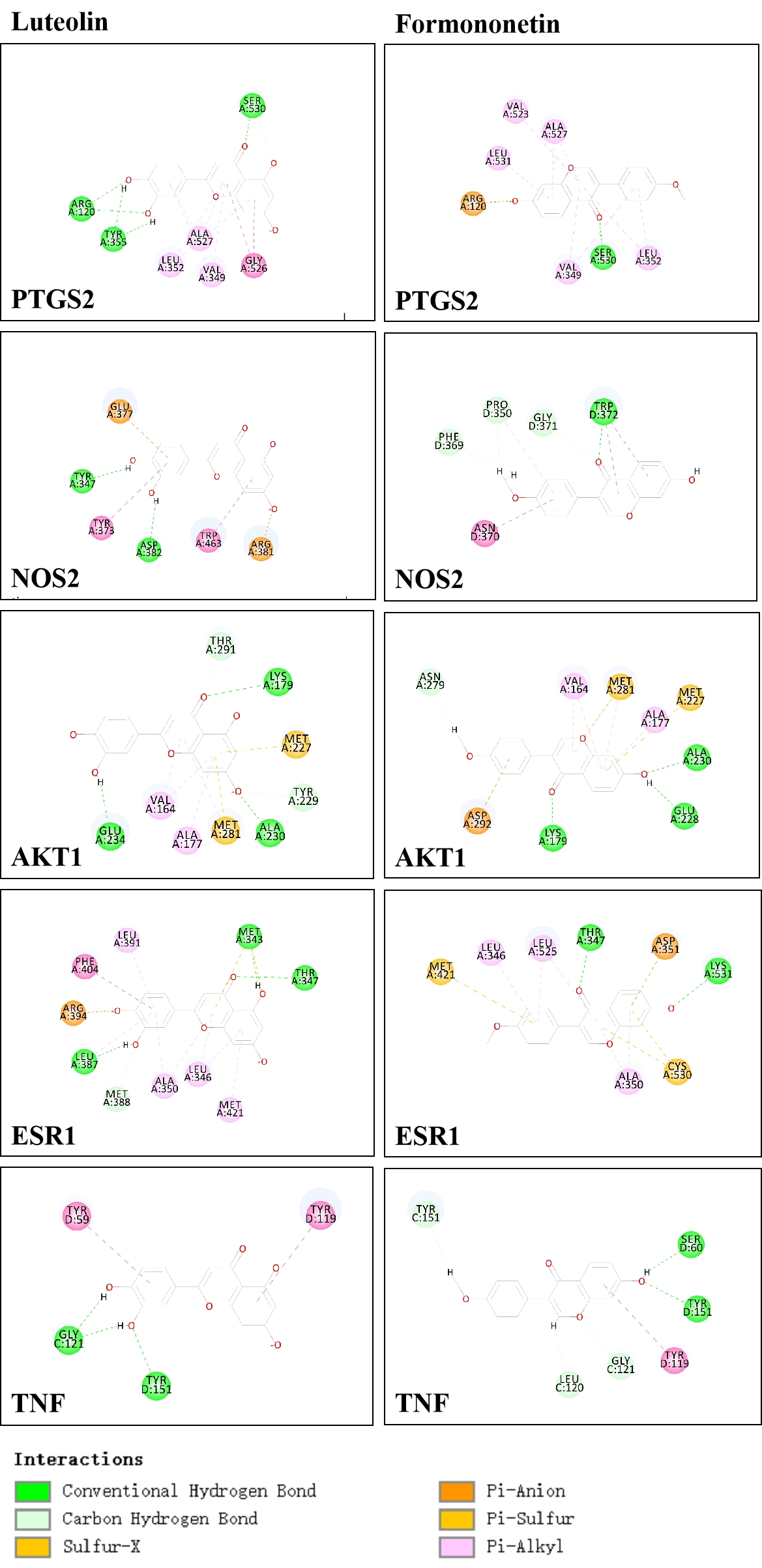


**Figure S1.** The 2D interaction diagrams of luteolin and formononetin with proteins


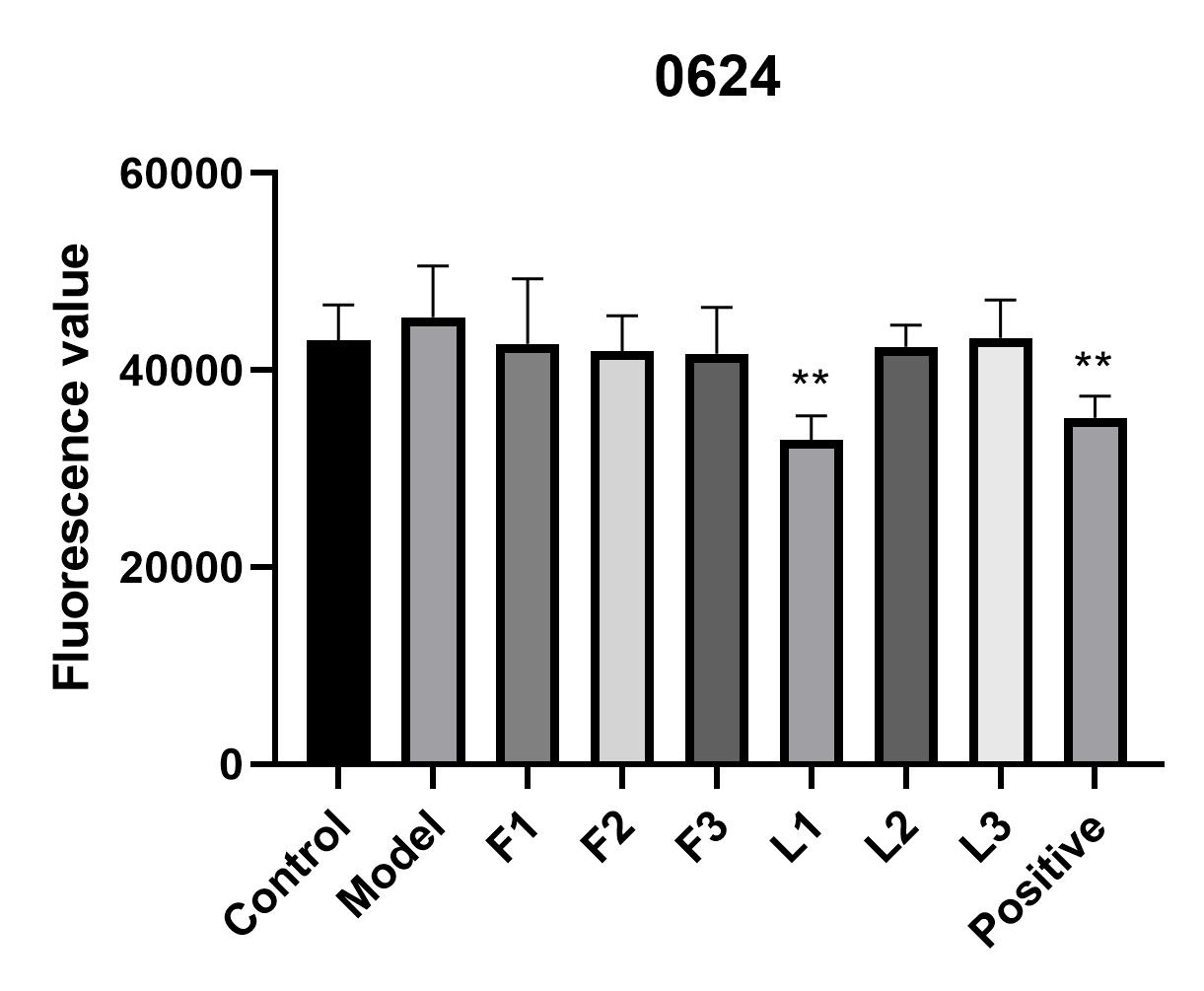


**Figure S2.** Effects of luteolin and formononetin on the ROS generation induced by HG (25mM/mL)

(F1-3: formononetin 40, 20, 10 µM; L1-3: luteolin 20, 10, 5 µM)


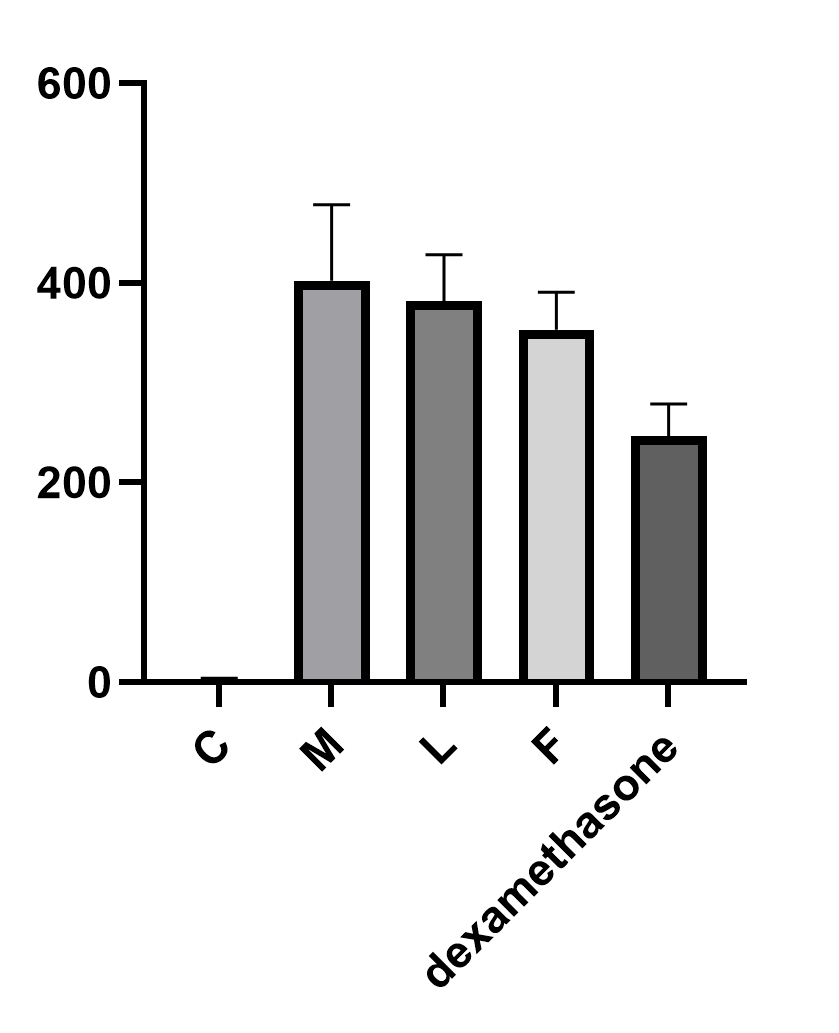


**Figure S3.** Dual‑luciferase reporter gene assay of luteolin and formononetin

(C: Control; M: Model; L: Luteolin; F: Formononetin)

**Table S1.** Comprehensive Interactions between the protein and luteolin

| PROTEIN | hydrogen bond interactions | π−π/π−alkyl interactions | -CDOCKER  energy | -CDOCKER interaction energy |
| --- | --- | --- | --- | --- |
| PTGS2 | Arg120, Tyr355, Ser530 | Val349, Leu352, Ala527 | 39.8 | 43.3 |
| NOS2 | Tyr347, Asp382 | Tyr373, Trp463 | 40.0 | 39.8 |
| AKT1 | Lys179, Ala230, Glu234 | Val164, Ala177 | 45.0 | 44.4 |
| ESR1 | Met343, Thr347, Leu387 | Leu346, Ala350, Leu391, Met421 | 43.6 | 42.9 |
| TNF | Gly121, Tyr151 | - | 32.4 | 31.9 |

**Table S2.** Comprehensive Interactions between the protein and formononetin

| PROTEIN | hydrogen bond interactions | π−π/π−alkyl interactions | -CDOCKER  energy | -CDOCKER interaction energy |
| --- | --- | --- | --- | --- |
| PTGS2 | Ser530 | Val349, Leu352, Val523, Ala527, Leu531 | 34.2 | 50.4 |
| NOS2 | Trp372 | Asn370 | 24.7 | 38.5 |
| AKT1 | Lys179, Glu228, Ala230 | Val164, Ala177 | 23.7 | 34.3 |
| ESR1 | Thr347, Lys531 | Leu346, Ala350, Leu525 | 23.2 | 33.9 |
| TNF | Ser60, Tyr151 | - | 15.3 | 25.2 |
